# Pain and the Brain: A Systematic Review of Methods, EEG Biomarkers, Limitations, and Future Directions

**DOI:** 10.3390/neurolint17040046

**Published:** 2025-03-21

**Authors:** Bayan Ahmad, Buket D. Barkana

**Affiliations:** The Signals Research Lab, Department of Biomedical Engineering, The University of Akron, Akron, OH 44325, USA; bbarkana@uakron.edu

**Keywords:** pain, brain, EEG, biomarkers, review

## Abstract

**Background:** Pain is prevalent in almost all populations and may often hinder visual, auditory, tactile, olfactory, and taste perception as it alters brain neural processing. The quantitative methods emerging to define pain and assess its effects on neural functions and perception are important. Identifying pain biomarkers is one of the initial stages in developing such models and interventions. The existing literature has explored chronic and experimentally induced pain, leveraging electroencephalograms (EEGs) to identify biomarkers and employing various qualitative and quantitative approaches to measure pain. **Objectives**: This systematic review examines the methods, participant characteristics, types of pain states, associated pain biomarkers of the brain’s electrical activity, and limitations of current pain studies. The review identifies what experimental methods researchers implement to study human pain states compared to human control pain-free states, as well as the limitations in the current techniques of studying human pain states and future directions for research. **Methods**: The research questions were formed using the Population, Intervention, Comparison, Outcome (PICO) framework. A literature search was conducted using PubMed, PsycINFO, Embase, the Cochrane Library, IEEE Explore, Medline, Scopus, and Web of Science until December 2024, following the Preferred Reporting Items for Systematic Reviews and Meta-Analysis (PRISMA) guidelines to obtain relevant studies. The inclusion criteria included studies that focused on pain states and EEG data reporting. The exclusion criteria included studies that used only MEG or fMRI neuroimaging techniques and those that did not focus on the evaluation or assessment of neural markers. Bias risk was determined by the Newcastle–Ottawa Scale. Target data were compared between studies to organize the findings among the reported results. **Results**: The initial search resulted in 592 articles. After exclusions, 24 studies were included in the review, 6 of which focused on chronic pain populations. Experimentally induced pain methods were identified as techniques that centered on tactile perception: thermal, electrical, mechanical, and chemical. Across both chronic and stimulated pain studies, pain was associated with decreased or slowing peak alpha frequency (PAF). In the chronic pain studies, beta power increases were seen with pain intensity. The functional connectivity and pain networks of chronic pain patients differ from those of healthy controls; this includes the processing of experimental pain. Reportedly small sample sizes, participant comorbidities such as neuropsychiatric disorders and peripheral nerve damage, and uncontrolled studies were the common drawbacks of the studies. Standardizing methods and establishing collaborations to collect open-access comprehensive longitudinal data were identified as necessary future directions to generalize neuro markers of pain. **Conclusions**: This review presents a variety of experimental setups, participant populations, pain stimulation methods, lack of standardized data analysis methods, supporting and contradicting study findings, limitations, and future directions. Comprehensive studies are needed to understand the pain and brain relationship deeper in order to confirm or disregard the existing findings and to generalize biomarkers across chronic and experimentally induced pain studies. This requires the implementation of larger, diverse cohorts in longitudinal study designs, establishment of procedural standards, and creation of repositories. Additional techniques include the utilization of machine learning and analyzing data from long-term wearable EEG systems. The review protocol is registered on INPLASY (# 202520040).

## 1. Introduction

Every person experiences some form of pain throughout their life. Upwards of 51.6 million Americans live with chronic pain [1], and 1 in 10 people worldwide are diagnosed with chronic pain annually [2]. Pain can arise in many different scenarios, including injuries [3]; diseases or conditions such as peripheral neuropathies, inflammation, and immune system disorders; damaged muscles, bones, or connective tissue; cancers, headache, migraine disorders, and more [3,4,5]; and surgical procedures [6]. It holds significance in guiding physicians in patient diagnosis, monitoring, and treatment. It also serves as an indicator for affected areas through pain receptors that relay signals to the spinal cord and further to the brainstem [7]. Pain intensity has been associated with disease activity [8], suggesting that it can estimate urgency and danger levels. Thus, pain can be considered a biomarker for measuring the presence or progress of an injury [9]. However, it is important to note that not all diseases and conditions are accompanied by pain. Indirect relationships between pain and disease can also exist. In such cases, pain intensity or duration may not be a reliable way of tracking disease progress or severity.

The International Association for the Study of Pain (IASP) has defined pain as an unpleasant experience relating to or resembling potential or actual tissue damage. It consists of both sensory and emotional components that can be affected by personal and environmental influences [10]. The association identifies several types of pain, including neuropathic, nociceptive, and neoplastic. Neuropathic pain is a result of abnormality, trauma, or disease of the somatosensory nervous system [10]. Nociceptive pain typically results from tissue damage, generally starting as acute pain, but it is susceptible to becoming a chronic condition [3]. In cases where the source of nociceptive pain is seemingly unidentifiable, it is referred to as nociplastic pain [10]. Any of these pain types can present as either acute or chronic. Acute pain refers to pain that is usually due to a temporary condition, such as an injury. This type of pain generally does not last long, but in some cases, the pain persists well after three months [3]. It can continue as long-term pain, otherwise known as chronic pain, which persists or repeatedly re-occurs for more than three months to half a year [11]. Approximately 28 to 61 percent of patients who experience acute pain develop chronic pain [12].

**Figure 1:**
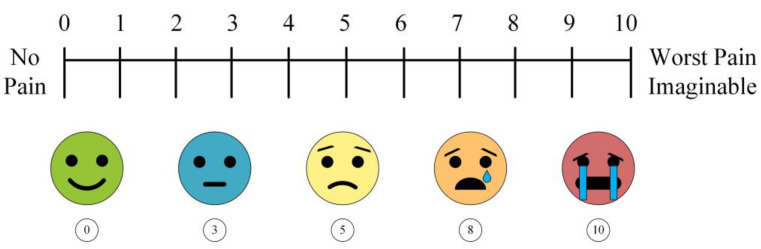
An example of a visual analog: Patients and participants commonly rate their pain on a scale of 0 to 10, where “0” implies no pain and “10” represents the worst pain imaginable. Visual markers are used as scale references.

There are several branches of chronic pain, including primary, cancer-related [5], postsurgical [6], post-traumatic, neuropathic [3], headache, orofacial, visceral, and musculoskeletal [11]. All of these can greatly restrict daily functional activities and quality of life. Pain in clinical settings is measured through verbal communication or self-reported techniques such as the Visual Analog Scale (VAS) and its variations [13]. An example is shown in Figure 1. Generally, this process consists of the patient rating their pain on a scale of visual intensities or numerical scores. One of the main concerns with these techniques is that the pain is subjectively assessed. Differences in individual perception are not accounted for and are applied on a generic scale. Thus, the reliability of these methods is low [13]. In addition, nonverbal patients or those with communication limitations, such as infants, toddlers, and those with certain disorders, cannot properly convey their pain intensity. For instance, physicians must rely on behavioral, physiological, and biological characteristics in the case of infants [14].

Current treatments include pharmacological methods, physical and psychological therapies, electrical stimulation, and surgery [15]. Most of these techniques serve as temporary solutions. Prescription medications such as opioids are often used to relieve pain. However, as the body’s tolerance increases, opioids lose their efficacy, resulting in dose increases over time. Patients also have a risk of addiction [16,17]. These dangers highlight the importance of proper pain diagnosis to offer the best treatment and develop interventions with minimum side effects. Another option is neuromodulation techniques, such as Spinal Cord Stimulation (SCS) [18]. This consists of applying electrical stimuli to certain areas of the spinal cord as a form of pain therapy. However, non-invasive techniques only serve as a temporary solution [19]. There is a great clinical need for pain treatment. There is ongoing research on identifying [20,21] and altering [22,23] pain perception as well as investigating biomarkers [24,25,26] for more accurate and effective pain assessment for a wider range of populations. Pain comprises two main components: sensory–discriminative and affective–motivational [27]. The sensory dimension encompasses spatial and temporal parameters, describing pain intensity [27]. The affective dimension embodies unpleasant pain; it urges a person to seek safety or protection [27]. Pain is perceived when the transduction of the nerve endings by a stimulus is transmitted to the brain. The transduction process is facilitated by sodium channels that translate the stimulus into electrical signals, while the conduction process transfers the signal to the central nervous system [28].

Researchers use an array of bio-signals to investigate the mechanisms and characteristics of pain by comparing data from pain and no-pain subjects [29,30,31,32]. Additionally, pain level responses are explored between participants [33], typically to classify pain states [34,35,36]. Variation in pain intensity is commonly attributed to the progression or severity of a disease [8,37], such as osteoarthritis (OA) [38]. It can also be due to physiological, emotional, and cognitive differences between individuals, since pain perception is subjective [39]. Ultimately, higher injury severity [40,41] and greater disease activity [8,42] are associated with higher subjectively reported pain levels. Some of the most common biodata collection methods for pain are electroencephalogram (EEG) measurements and functional imaging techniques, such as functional Magnetic Resonance Imaging (fMRI), which provide insights into brain activity. EEGs use electrodes placed on the scalp to measure neural oscillation frequencies and event-related potentials (ERPs) [43]. EEG frequency bands are common features derived for frequency-domain analysis; they are classified into five major ranges: delta (1–3.9 Hz), theta (4–7.9 Hz), alpha (8–12.9 Hz), beta (13–30 Hz), and gamma (30–100 Hz) [44]. fMRI utilizes the changes in blood flow due to neuron firing to identify active sites in the brain [45]. When brain regions are active, metabolic activity increases their demand for blood supply [46]. These neural features are analyzed to uncover how pain is represented in the brain. These methods are also used to capture how these features change between pain states and variations in intensity [45,46].

In recent years, advances in EEG device technology have made it possible to non-invasively collect high-quality data regarding the temporal activations of brain regions. In regard to the activation sources, high-density EEGs (hdEEGs), that have 64 or more electrodes, with standardized low-resolution brain electromagnetic tomography (sLORETA) and independent component analysis (ICA) algorithms, provide high accuracy. Accurate electrode placement is critical since signal interpretations and source localizations are based on the electrode locations. Incorrect or inconsistent electrode positioning can lead to the mislocalization of brain activity. Small deviations can result in errors in functional mapping, connectivity analysis, and clinical diagnoses. A person’s scalp acts as a signal-blurring zone, where electrical currents are altered as they pass through the resistance between the cortical layer and the electrode [47]. With a higher number of electrodes, it becomes possible to cluster groups of signals and allocate them to more-specific brain regions [48]. This becomes increasingly difficult and unreliable for low-density EEGs (ldEEGs) (32 electrodes or fewer), where electrodes are spaced much farther apart [49]. Thus, poor spatial resolution is a common limitation seen with ldEEG acquisition [50]. For this reason, using a high-density EEG system is a better option for higher precision. A trade-off of using hdEEGs lies in their preparation time. Each of the electrodes must be properly situated on the scalp, based on a system (e.g., 10–10 or 10–20 system), and the more electrodes, the longer it takes to prepare the participant for recording. This is also important when factoring different types of high-density (64, 128, and 256-channel) systems. A study noted that the most significant improvement occurred when increasing from 32 to 64 electrodes, while further increases in the number of channels yielded less-significant results [51]. Additionally, non-invasive ldEEGs can only record activity near the surface of the scalp [50]. Neural activations in the deeper parts of the brain become weaker as they reach the surface. This is why ldEEGs typically assess cortical activity [50]. Due to their poor spatial resolution, MRI and mathematical models are frequently used in conjunction with EEGs to perform source localizations [48]. EEGs are only able to provide information about neural activity at the time of the recording [52]. In cases where long-term effects are to be analyzed, multiple recording sessions over time are necessary. This poses a limitation on the clinical application of EEGs for pain rating. If a patient typically experiences severe pain but has a lower pain intensity at the recording time, the EEG—and consequently the pain rating—may not accurately represent the patient’s actual state of pain.

**Figure 2:**
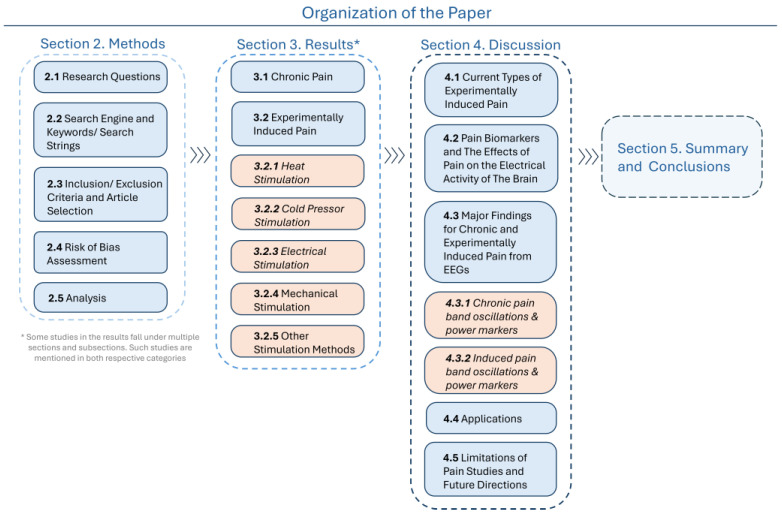
A flow diagram for the organization of the paper.

Due to the global prevalence and impact of pain, there is significant research interest in understanding the interactions between pain and brain activity using various modalities. Among these, EEGs have gained considerable attention in recent years. However, many studies adopt unique evaluation and analysis criteria, which poses challenges for cross-study comparisons. This work systematically reviews the existing methods and identifies neurological markers such as brain activity, neural processes, or neurological states associated with pain and their associated limitations. The work is organized in the following order. Section 2 states the research questions and the Preferred Reporting Items for Systematic Reviews and Meta-Analysis (PRISMA) diagram. Section 3 presents the results regarding the studied pain types and their markers in the brain. Section 4 discusses the reported findings and the study’s limitations. The organization is outlined in Figure 2.

## 2. Materials and Methods

### 2.1. Research Questions

We formulated three research questions using the Population, Intervention, Comparison, Outcome (PICO) framework structure [53]. The desired population was people in or subjected to pain-state conditions. The interest was pain biomarkers, while the context was EEG signals. Three constructed research questions were answered to gain insight into the existing studies’ methodologies, analysis, and limitations.

1. In humans, what experimental EEG approaches are being used to study pain states compared to pain-free control states, and what neurophysiological insights do they provide?
2. In individuals experiencing chronic pain, what electrical brain signal responses differ from those undergoing experimentally induced pain, as measured by neurophysiological techniques?
3. In studies investigating human pain states, what methodological limitations affect the reliability and interpretation of the findings?

### 2.2. Search Engine and Keywords/Search Strings

The keywords used to identify articles of interest were selected from the PICO components. The keywords were “Electroencephalography” or “EEG” and “Pain Biomarkers” or “Pain Biomarker”. These keywords were used to acquire broad results that focused on our topics of interest. We searched and reviewed the studies published in PubMed, PsycINFO, Embase, Cochrane Library, Scopus, IEEE Explore, and Web of Science databases until December 2024. Advanced searches were used to arrange the keywords into search strings. The strings consisted of (“Electroencephalography” OR “EEG”) AND (“Pain Biomarkers” OR “Pain Biomarker”). The total search results consisted of 592 articles.

Multiple search databases were used to prevent bias by ensuring the comprehensive coverage of the relevant literature and minimizing the risk of selection bias. PubMed focuses on biomedical sciences; PsycINFO specializes in psychology; Embase covers biomedical, pharmaceutical, and clinical medicine research; Cochrane strongly emphasizes clinical trials, interventions, and healthcare decision-making; and IEEE Xplore covers engineering and technology. Publication bias was reduced by including studies from different disciplines, regions, and databases.

### 2.3. Inclusion/Exclusion Criteria and Article Selection

Our inclusion and exclusion criteria were designed to ensure the relevance and quality of the studies analyzed. To be included, studies had to focus on collecting EEG data related to a chronic or experimental pain state and address at least one of the research questions. We included studies both with and without control groups, as long as they contributed to answering the review’s questions. Studies were excluded if they did not involve human subjects, did not utilize EEGs, did not assess pain states, assessed non-experimental acute pain, or failed to report on neuro-biomarkers of pain. Additionally, we excluded invasive EEG studies and those lacking essential details regarding the participant population, pain type, or study design.

Study relevance was assessed for each of the studies. We used a relevance scoring system similar to the systematic reviews in the literature [54,55,56,57], where two independent reviewers performed the research, read the titles/abstracts, and then scored each study. A relevance score of “0–2” was given to each article to quantify a grading scale based on our inclusion and exclusion criteria. In most cases, the process used in deciphering between the included and excluded studies was qualitative. The abstracts of 122 studies were manually assessed, and a score of “0” was given to studies that showed no relevance. A “1” was given to somewhat relevant studies, such as those that included some information on EEG signals and pain states. A “2” was given to studies that were highly relevant to the research questions.

### 2.4. Risk of Bias Assessment

Bias levels were determined considering the study limitation concerns presented in the Newcastle–Ottawa Scale [58]. The Newcastle– Ottawa Scale (NOS) was used by two independent reviewers (B.A. and B.B.) to assess the risk of bias in the included studies. The scale consists of three sections: Selection, Comparability, and Exposure. The Selection section includes four items, the Comparability section has two items, and the Exposure section comprises four items. Each item is rated with one star, reflecting the study’s methodological quality. Table A1 presents the study risk of bias assessment for each included study.

### 2.5. Analysis

The study data analysis included assessing the participants’ demographic information, study materials and methods, study features, reported results, and limitations. Descriptive statistics were used to report the results and compare the characteristics of different studies and the results between studies with specific focuses on pain induction/state methods and neural activity assessment. The same data were assessed for each study as was applicable. Meta-analysis was not performed, since the included studies were too heterogeneous because of the varying numbers of EEG electrodes, various pain stimulations and chronic pain sources, and experiment protocols.

## 3. Results

**Figure 3:**
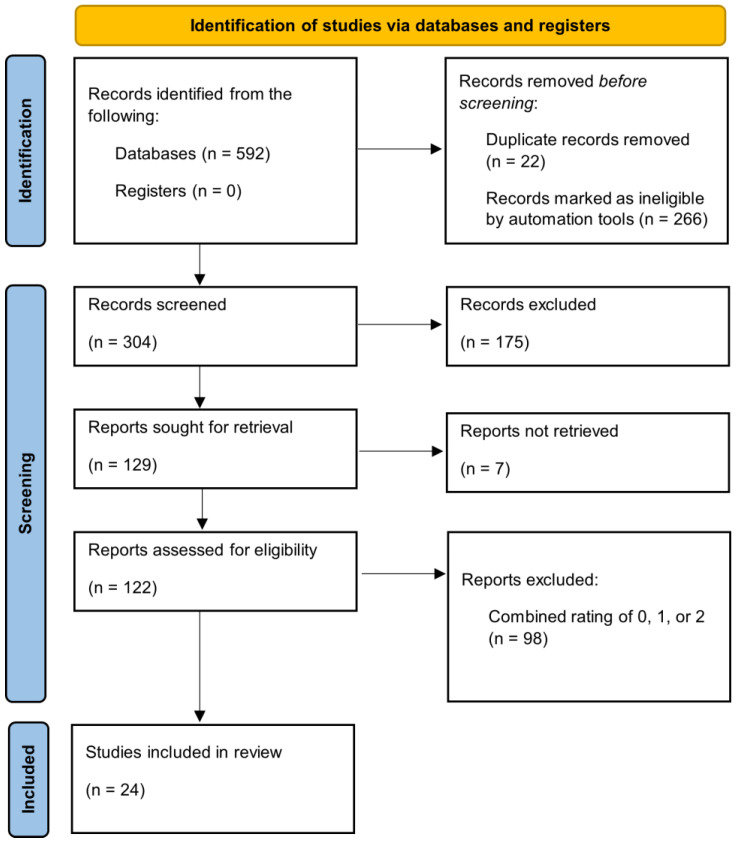
A Preferred Reporting Items for Systematic Reviews and Meta-Analysis (PRISMA) diagram, summarizing the flow from the initial number of article results, to the exclusions and inclusions, and finally to the final articles included in this review.

The keywords were central components of the research questions and any relevant article. Thus, the initial screening excluded articles that did not contain both keywords or were presented in a language that was not English (*n* = 266). Duplicates were then removed from the list of eligible articles (*n* = 22). An Excel file was created to compile the list of the remaining articles (*n* = 304). Only original research studies were used for evaluation; all other literature types, such as review and opinion papers, were removed (*n* = 175). Articles that were not retrieved were excluded (*n* = 7). Two independent reviewers manually screened the remaining 122 studies. After the relevance scoring and risk of bias evaluation, a total of 24 studies were included in the review. This systematic review complies with the PRISMA guidelines. Figure 3 shows the PRISMA diagram, summarizing the article selection, screening, and inclusion flow.

EEG data were recorded to track the neural activity resulting from a certain condition or lack thereof. Pain data were usually collected from chronic pain or experimentally induced pain states. In either case, EEGs were recorded to define pain responses, recovery periods, time intervals, source locations, or possibly pain prediction. This review analyzed 24 studies [59,60,61,62,63,64,65,66,67,68,69,70,71,72,73,74,75,76,77,78,79,80,81,82]. Fourteen of these consisted of chronic pain data analysis [59,60,61,62,63,72,73,74,77,78,79,80,81,82], and sixteen pertained to pain induction methods [64,65,66,67,68,69,70,71,73,74,75,76,78,79,81,82]. Often, studies used multiple stimulation methods [60,62,66,72,75,78,79,81,82] or subjected chronic pain participants to experimental pain [60,62,72,78,79,81,82] to investigate the potential relationships between pain induction modalities and to analyze how chronic pain subject processing differed from healthy control processing when subjected to external pain. Between the chronic pain and experimental pain studies, eight utilized heat pain [62,64,65,66,67,78,79,82], ten used cold-induced pain [60,62,68,69,70,71,72,79,81,82], three applied electric methods [59,66,73], two used mechanical stimulation [74,78], one used an algometric stimulus [81], and two studies utilized nerve growth factor [75,76]. Only one entirely male study was present [68]. The most common female participation percentage fell between 36% and 60%, with nine studies falling into this category [59,63,65,67,69,70,75,76,80]. The second most common, also with nine studies [61,62,66,72,73,77,78,79,82], fell between 61% and 90% female participant count. Three studies [64,74,81] fell below 35%, and one study [60] had greater than 90% female participants. One study did not mention this demographic information [71]. In addition, the typical study population criteria consisted of a wide age range, from 18 and 20 years old to above 60 years old. This range was most commonly seen, and was the case for fifteen studies [60,61,63,68,69,70,71,72,73,74,76,77,78,81,82]. Five studies [64,65,66,67,75] limited the adult range to mid-30s and 40s, while one study [59] started its inclusion age range at 40 years old. Only one study [62] focused on pediatric populations, starting at 10 years old. One study’s minimum age requirement was 16 years old [79], and one study did not disclose its age eligibility criteria but included both adult and later pediatric populations [80]. Regarding data analysis, twelve studies [61,63,65,67,68,73,74,76,77,80,81,82] based their results and conclusions on data from 21–50 participants. Five studies [60,64,66,75,79] based them on 51–80 participants, while another five studies [59,69,70,71,78] looked at data from 20 or fewer subjects. Only two studies [62,72] drew results from over 81 participants. The electrode count is an extremely important factor to consider when assessing findings. Higher-density caps will result in more precise source localization than lower-density ones. Fifteen studies [60,61,64,65,66,68,69,72,73,74,75,78,79,80,82] used high-density EEG acquisition, twelve of which had 63 or 64 active electrodes [61,64,65,66,68,73,74,75,78,79,80,82], and three had 128 electrodes [60,69,72]. Table 1 and Table 2 provide further information regarding study details, including equipment, methods, participant characteristics, features, and study limitations.

### 3.1. Chronic Pain

Chronic pain has been an area of interest for many years. With the high number of populations affected globally, there is research focused on evaluating the mechanisms of chronic pain processing. With this knowledge, better approaches to solutions can be explored. It has been reported that patients experiencing pain have changes in brain signal frequency, power, and speed, and those experiencing chronic pain tended to have a higher theta power and resting-state alpha power [55]. Chronic pain studies frequently collect resting-state EEG data to analyze how patients’ neural activity differs from those not in chronic pain conditions. Barbosa et al. [77] identified chronic neuropathic pain (CNP) biomarkers from resting-state EEGs. Their analysis was based on previously collected data from a study [83] in which data were collected from 32 CNP patients. Two 5 min EEG recordings were taken, one with the eyes open and the other with the eyes closed. They calculated the resting-state spectral power for different frequency bands and brain regions. The reported alpha, beta, and delta oscillations in the central, frontal, and parietal brain regions were related to pain scores. Similar associations were seen for pain scores and interference in general activity and mood.

Two studies performed by Case [80] used EEGs and fMRI acquisition to study the differences in patients with sickle cell disease compared to healthy controls. Sickle cell disease is often associated with chronic pain, necessitating a deeper understanding of its neurophysiological processing in order to develop effective pain management strategies. In the resting-state EEG study, 24 patients with sickle cell disease (SCD) and 14 healthy controls were recruited, with 20 SC participants in the final analysis. Power spectrum and source localization analyses revealed increased power in low-frequency ranges, including the delta, theta, and alpha bands. Particularly, the beta2 and theta bands showed significant differences between SCD patients and healthy controls, highlighting potential neural signatures of pain in sickle cell disease. EEG source imaging in the study revealed increased theta activity in the prefrontal cortex, left Rolandic operculum, left insula, left putamen, and caudate nucleus in the SCD group, along with elevated beta2 activity in the prefrontal cortex, anterior cingulate cortex, right superior temporal gyrus, and right caudate nucleus. Additionally, the theta-band peaks differed between the two groups, and a center of gravity analysis indicated gamma-band variations. These findings suggest that chronic pain and, consequently, SC severity are associated with higher theta power. In a simultaneous EEG-fMRI study, data were collected from 15 SCD patients and 15 healthy controls; however, the final analysis included data from 11 SCD participants and 13 controls. Resting-state EEGs were recorded both inside and outside the fMRI scanner, with each session lasting 20 min under an eyes-open condition. The results indicated that, compared to the controls, the SCD group exhibited reduced activity in the default mode network (DMN) and executive control network. Conversely, SCD patients showed increased connectivity in resting-state networks. Additionally, higher frequency modulation was observed in the cerebellum, posterior precuneus/posterior cingulate cortex (PCC), medial frontal cortex, inferior and superior parietal regions, superior frontal regions, and intraparietal sulcus of the SCD patients.

Another resting-state EEG study [81] investigated the resting-state EEGs of 39 spinal cord injury patients and their relationships to conditioned pain modulation responses and demographic and clinical variables. The EEG acquisition consisted of a 20 min recording while the participants’ eyes remained closed. QST was used for the conditioned pain modulations, specifically the pain pressure and cold-water stimuli. The central, parietal, and frontal brain regions were used to calculate the average power spectra. Spinal cord injury (SCI) patients with pain showed less alpha power compared to SCI patients with no pain. In all the target regions, the pain group displayed greater theta power, as well as upper beta power and sub-band power over the parietal region. Similarly, increased subjective pain ratings were related to lower alpha and beta powers. Other CPM factors were assessed and correlated to the resting-state EEGs. The study performed area-under-the-curve (AUC) analysis and reported it as a potential pain marker for chronic pain, since the AUCs were higher for the alpha and beta bands in specific brain regions: alpha band (AUC = 0.81 in central, frontal, and parietal areas), beta band (AUC = 0.78–0.83 in parietal areas).

Investigating the clinical and physiological predictors of brain oscillatory activity in patients with fibromyalgia (FM), ref. [79] assessed the EEG signatures of chronic pain under resting and motor task conditions. This cross-sectional study was based on another study, investigating the mitigation of FM symptoms with transcranial direct stimulation and aerobic exercise [84]. A total of 78 participants diagnosed with FM were recruited, but only 73 participants’ data were viable for EEG analysis. Conditioned pain modulation responses were also assessed. The resting state consisted of a 5 min recording with the eyes open. The motor phase consisted of watching a video of a clenched hand for the observational component, clenching their hand for the motor task, and imagining clenching their hand for the imagery task. Event-related spectral perturbations were assessed for each of the three motor conditions. Frequency bands and sub-band relative powers were calculated for the frontal, central, and parietal brain regions. Further clinical data were used to characterize the EEG patterns. A highlight was that the resting-state components showed a negative relation between the beta band and pain intensity but a positive correlation with fibromyalgia duration. Another cross-sectional analysis [82] of the same study [84] assessed neurophysiological patterns between fibromyalgia pain, conditioned pain modulation, and transcranial magnetic stimulation. The researchers collected 10 min resting-state EEGs—5 min eyes open and 5 min eyes closed—from 26 fibromyalgia patients. Similar motor tasks were conducted. The power analysis revealed that pain intensity was inversely correlated with the frontal, central, and parietal alpha powers. Similar results were found for the beta power in the parietal region.

In many instances, studies used additional stimulation to assess the effects of chronic pain on neural functioning or to compare brain responses from chronic pain patients and healthy controls. The first was the case for a 2023 study by Vanneste and De Ridder [59], where the objective was to observe the relations of the imbalance model in chronic pain. This model suggested that chronic pain is a result of an imbalance between pain-inducing and pain-inhibiting signals from the three main processing pathways, resulting in persistent pain. Based on this model, a decrease in chronic pain by way of Spinal Cord Stimulation (SCS) would be reflected in the neural oscillations. The study utilized both a chronic pain population and electrical stimulation. However, the electrical stimulation was not used for pain induction. Fifteen patients between the ages of 40 and 67 who were deemed eligible for SCS underwent surgery to implant a Lamitrode 88, an internal pulse generator (IPG) programmed to achieve maximum pain suppression through its burst mode. The IPG was stimulated at a lower amplitude for a longer pulse period. EEGs were recorded to observe neural frequencies. Electromagnetic tomography was used to localize the EEG signals on the cortex. Comparing pre- and post-stimulation values, the pregenual anterior cingulate cortex (pgACC) had a significant decrease in its theta-band current density. The dorsal anterior cingulate cortex (dACC) had a significant decrease in both alpha and beta current density. The gamma and theta bands’ current density decreased for the left and right SSC. The functional connectivity of the alpha band increased significantly. For the theta band, the left somatosensory cortex (SSC) significantly decreased its communication with the pgACC, but pgACC to left SSC communication increased. Comparable results were observed for the right SSC and pgACC. For alpha, a significant increase was observed in the pgACC to the left and right SSC and the dACC. A significant reduction in theta–gamma coupling for the SSC and alpha–beta coupling for the dACC was observed. Overall, the results support the idea that chronic pain is an imbalance between pain-input and pain-suppression pathways. It also suggests that burst stimulation could work to counteract this imbalance. By comparing the differences between pain and pain-relieved states, changes in the frequency bands were noted, ultimately highlighting a possible direction for solutions [59].

Ding et al. [78] assessed neural markers in chronic pain resting states and pain stimulation conditions compared to healthy controls to address the question of how neural responses to stimulation differ for chronic pain patients. Central sensitization causes chronic pain when sensitivity is heightened due to several factors that increase neuron firing, resulting in painful sensations in response to normal stimuli. This makes it a unique type of chronic pain, as it does not proportionally correlate with stimuli parameters [85]. Eight central sensitization patients and eight healthy controls were first subjected to a resting condition consisting of 2 min with the eyes open and 2 min with them closed. This was followed by the pain stimuli component, which consisted of thermal and mechanical pinprick QST stimuli. The results showed that chronic pain patients had more substantial alpha frequencies but significantly lower PAFs when their eyes were closed compared to the control participants. Additionally, chronic pain participants displayed lower theta and beta activities. However, their relative alpha power decreased while the theta and beta powers increased during the QST. This relationship was inverted for the healthy controls. The study highlights power ratios and PAF as potential biomarkers for central sensitization.

Another study assessed the correlations of sociodemographic and clinical variables to EEG neural oscillations in patients with chronic knee osteoarthritis (KOA) pain. EEG recordings were collected from 66 patients over the age of 18 with KOA. In addition to transcranial magnetic stimulation, pressure pain thresholds and conditioned pain modulation with cold water were used, similar to the quantitative sensory assessments and experimentally induced pain procedures described in Section 3.2. The results showed that higher pain intensity and KOA severity levels correlated to increased frontocentral beta and high beta power and an overall decreased theta activity. Alpha and delta frequencies are associated with greater cortical inhibition. Increased delta and theta power relate to poor cognition and age, while greater beta and alpha powers correlate to decreased motor function and the extreme degeneration of joints. For neuropathic pain, increased theta oscillations were observed, but the results for KOA showed a correlation between pain and decreased theta oscillations. Higher beta powers were associated with greater KOA severity and self-reported pain during functional activities. A positive correlation was observed between motor-evoked potentials and alpha oscillations. The work reported higher delta waves associated with increased intracortical inhibition [60]. In 2023, Simis et al. [72] investigated the factors that may predict dysfunctional conditioned pain modulation (CPM) in KOA patients. The purpose was to better understand the pain characteristics in KOA patients and to design treatment plans unique to the patients. The demographic information, pain scores, CPM responses, and neurophysiological responses of 85 subjects with KOA were collected. The neural variables consisted of transcranial magnetic stimulation using the Magstim Rapid stimulator and EEG recordings deriving absolute and relative powers. Each participant underwent the same pain-related variable assessment, which is presented in [60]. A 128-channel EGI system was used for EEG data collection. The results showed that there was a significant negative correlation between Western Ontario and McMaster Universities Osteoarthritis Index (WOMAC) scores and CPM. Also, high Berg Balance Scale (BBS) scores had an apparent relationship with CPM in the participants. The relative delta powers in both the frontal and central areas were negatively correlated to CPM [72].

Information on pain-free states is just as beneficial. It provides a comparison and serves as a baseline. This can be obtained by relieving existing pain in a group or comparing a pain-state group with a pain-free group. Heitmann et al. used the first technique. Their goal was to explore biomarkers of chronic pain and treatment for potential monitoring over time. Data from 50 clinically diagnosed chronic pain patients were observed before and six months after interdisciplinary multimodal pain therapy (IMPT). EEG recordings and standardized clinical questionnaires were collected pre- and post-IMPT. The IMPT program consisted of 20 days of treatment with medical and behavioral therapy. Pain intensity, physical functioning, and emotional functioning data were collected for the clinical measures. Data from 41 patients were analyzed. The dominant peak frequency, neural oscillation power, and functional connectivity were analyzed from the EEG results. No significant correlation existed between the clinical measures and dominant peak frequency or neural oscillation power. Between the local network and functional connectivity, there was a significant positive correlation in the brainstem and cerebellum between local beta-band connectivity changes and pain-related disability. Although insignificant, the results strongly suggested a negative correlation between the global efficiency (gEff) in the theta band, pain intensity, and pain-related disability. Overall, their results suggested that the global network in the theta band could be used for long-term chronic pain monitoring [61].

The pediatric population is a vulnerable group in need of pain biomarkers. Some subpopulations are not able to interpret or relay the amount of pain they are feeling and where it is located. Ocay et al. [62] set out to investigate the brain activity and connectivity of pediatric patients with chronic musculoskeletal pain. They hypothesized that the EEG recordings of the patients would reflect at-rest conditions and thermal pain stimuli differently than in healthy controls of the same age. Data from 151 chronic pain patients and 45 healthy control patients were recorded. Each participant completed the tonic heat stimulus and cold pressor task referenced in Section 3.2, and their pain intensities were recorded. The spectral power, peak frequency, permutation entropy, weighted phase-lag index, directed phase-lag index, and node degree were extracted from the EGGs. Data from 142 chronic patients and all the healthy controls were analyzed. The results show that the only demographic characteristic significantly correlated to neural power is an age-related decrease in resting global theta power. The chronic pain patients exhibited increased delta and beta global powers. Overall, the results implied increased thermal pain sensitivity in chronic pain patients. Chronic pain patients also showed increased permutation entropy during the heat stimulations and network functional connectivity during the cold pressor test. The results showed differences between the chronic pain patients and the healthy controls [62]. Further investigation can help to identify the underlying mechanisms of the pain associated with these conditions in the pediatric population and present support for pain sensitivity monitoring.

Another cause of chronic pain is chronic pancreatitis. This is why the objective of a study performed in 2013 by De Vries et al. [63] was to search for pain biomarkers through resting-state EEGs of subjects with chronic pancreatitis (CP) through the alpha range. EEG recordings were collected from 16 patients with chronic pancreatitis experiencing abdominal pain and 16 healthy age-, sex-, and education-matched participants. The study reported that the peak alpha frequency (PAF) decreased in the CP group compared to the healthy controls. This is consistent with other studies reporting a slowing of neural oscillations. Also, a more significant reduction in PAF was associated with a longer pain period. This could be a potential marker for disease progression. The results also reported that the PAF was primarily located in the parietooccipital region [63].

Two of the chronic pain studies [73,74] focused on eliciting induced pain responses; consequently, they are discussed in Section 3.2.

### 3.2. Experimentally Induced Pain

Quantitative sensory testing (QST) is a collection of psychophysical assessments that are used to evaluate a participant’s somatosensory processing. It encompasses thermal, mechanical, vibrational, and pressure tests. This procedure allows for a standardized method of activating participant somatosensory nerve fibers to assess their functionality, revealing pain processing mechanisms [86]. The German Research Network on Neuropathic Pain (DFNS) testing protocol is one of the most common standardized protocols, but researchers often design their own procedures that have similar techniques [86]. Often, studies choose to experimentally induce pain directly with one of these methods. Many researchers are investigating how the brain responds to pain-related stimuli, mostly to assess the brain’s response to such conditions and characterize biomarkers for acute pain. Another reason is to translate their results to chronic and related pain. In most cases, participants were subjected to a form of experimentally designed pain, and EEG signals were recorded and analyzed.

#### 3.2.1. Heat Stimulation

The DFNS QST thermal tests include cold and warm detection thresholds and pain thresholds. They also encompass a thermal sensory limen. A thermal sensory thermode device is utilized for this procedure, activating the Aδ and C fibers. The mean of three threshold temperature recordings is used for measurement analysis [86]. Often, studies will utilize a similar technique to collect heat pain measurements.

Ocay et al. [62] utilized this heat stimulus method on their chronic pain subject group by using a warm thermode on the right volar forearm. They induced a 50% pain intensity for a total of 120 s. Furman et al. [64] utilized both heat stimuli and capsaicin to investigate whether pain-free PAFs were related to prolonged pain sensitivity. This was achieved by comparing, during two separate visits, the resting alpha-band frequency and pain sensitivity of 61 healthy individuals. The pain conditions included phasic heat pain (PHP) by way of the Contact Heat Evoked Potential Stimulator (CHEPS) and capsaicin heat pain (CHP) using a capsaicin paste. The PHP was induced through five intervals of the heat stimulus for 1 min. The CHP induction consisted of increasing the pain sensitivity of the participant. Continuous pain ratings were collected during the EEG recordings. A total of 57 participants from the first visit and 43 from the second visit had their data analyzed. The results showed that PAF was inversely related to both prolonged pain conditions and that this relation was dependable over extended periods. The study showed that PAF can be a reliable marker for pain sensitivity and possesses possibilities for clinical applications [64].

Ding et al. [78] performed QST heat stimulation with 10 pulses of a 45 °C calibrated thermode on the volar of the non-dominant forearm. Each stimulus lasted half a second, with a 2.5 s rest between pulses. Two chronic pain studies [79,82] utilized heat stimulation during a conditioned pain modulus response test. Both determined the pain-60 temperature for each participant, which is the temperature at which a pain score of 60 out of 100 is given in response to the stimuli. Both studies applied the heat stimulus on the right forearm for 30 sec. This was then repeated during the cold pain modulation. Pain ratings were collected both times.

Another study aimed to understand the transient and sustained brain processing mechanisms involved in relating nociception to the perception of pain [65]. To achieve this, the study recorded EEGs from 48 healthy participants while administering a CHEPS stimulus. With three different durations and two intensity levels, there were a total of six duration–intensity combinations. The stimuli were divided into six sections, each with 30 stimuli (5 for each combination type). The results demonstrated that the brain responded to pain and that pain perception was correlated or influenced by the stimuli’s duration and intensity. The results indicated a low frequency from the insula and anterior cingulate cortex and an alpha event-related desynchronization from the sensorimotor cortex, which correlated to the alpha frequency oscillations [65].

Beck et al. [66] assessed the ability of EEG data of nociceptive laser stimuli to differentiate between the intensity of stimuli and the participants’ subjective pain. EEG data from 77 healthy individuals experiencing radiant heat stimulation were collected. In one session, participants distinguished between radiant heat stimuli intensities. In the other session, participants distinguished between two transcutaneous electrical stimuli intensities as the control. The electrical stimulation methods are outlined in Section 3.2.3. The data analysis was based on 57 of the 77 subjects. The results indicated that gamma-band oscillations (GBOs) were higher for the laser than electrical stimuli. While the laser-induced GBOs remained unchanged, the electrical stimuli caused a decrease in GBO. Overall, the changes observed were minimal to negligible. Additionally, the N2 and P2 vertex waves were more sensitive to stimulus intensity in response to the laser stimuli than to the electrical stimuli, with N2 also reflecting subjective pain intensity perception. These findings suggest that laser-evoked potentials provide greater insight into nociceptive processing than somatosensory-evoked potentials [66].

A study conducted by Nuñez-Ibero et al. [67] looked to design a device for pain biomarker identification. The objective was to record brain activity and hemodynamic interactions during heat stimulation. A device was designed to apply a heat stimulus while simultaneously recording EEG and photoplethysmography (PPG) readings. The study consisted of 35 healthy participants. A heat pain threshold was determined for each participant, and temperatures of 90% of the threshold were applied as the pain stimuli for the recording. The PPG results showed that thermal pain was reflected by a decrease in heart response entropy. The EEG results showed neural oscillation in the beta and theta bands resulting from pain, which was apparent in both the left and right hemispheres. Statistically significant differences in the EEG (both right and left hemispheres) and PPG spectral entropy were identified between the pain and control groups, suggesting that spectral entropy can differentiate pain states [67].

#### 3.2.2. Cold Pressor Stimulation

DFNS QST uses the same method for both cold and heat pain. However, cold pain is typically induced through the cold pressor procedure. This is a test where a participant submerges their hand, arm, or foot into an ice-water bath. The technique also has many variations regarding its protocol. Many experimenters will adjust the duration, the water temperature, the equipment, or the submerged limb.

Five studies [60,72,79,81,82] utilized this method in their chronic pain studies during their conditioned pain modulation procedure. Their participants were instructed to place their hand—usually the non-dominant hand—into water set at 10–12 °C for a total of 30–60 s, then pain ratings were collected for each of the studies. Another study [68] investigated and compared central sensitization clinical variables, pain response, and the mechanism of gamma-aminobutyric acid (GABA)-dependent inhibition in athletes and non-athletes during cold pain stimulation. Their hypothesis was that the athletes would experience decreased pain perception and central sensitization (CS) features. EEG recordings were collected for all 27 healthy athletes and 27 age- and sex-matched non-athlete participants during the cold pressor test. The analyzed participant data consisted of 26 athletes and 24 non-athletes. Pain perception and pain sensitivity were assessed through a numerical rating scale. The pain perception time reflected the resistance to pain. The pain response revealed that the athletes did not significantly experience less pain sensitivity. Also, CS index scores were negatively correlated to pain perception time. The results indicated that the CS components influenced pain resistance in non-athletes. The results regarding changes in EEG markers suggest that the increase in lower beta as a response to pain stimulation was inversely related to CS. This mechanism could be a counteractive measure of pain in non-athletes. The study verified that pain response mechanisms and biomarkers differed between the athletes and non-athletes [68].

Chouchou et al. [69] assessed the differences in gamma and alpha spectral power in both pain conditions and pain-mimicking facial grimaces. Gamma spectral power is highly influenced by muscle contraction due to pain. Therefore, it is difficult to distinguish gamma signals due to pain from those due to muscle movement. The study collected pain intensity and EEG recordings from 14 healthy participants subjected to two separate conditions. One of the conditions was muscle contraction, and it consisted of the participants mimicking the facial expressions resulting from pain for 10 s. The pain condition consisted of the cold pressor test. The results showed that the gamma power topography for both the pain and grimace conditions was similar, while the alpha power during the pain conditions did not match that of the grimace condition. This suggests that the alpha power is more resistant to pain-related muscle responses and can be a stronger pain biomarker [69].

Rather than looking for changes in EEG signals at the time of stimulation, one study focused on investigating how the brain responds following a painful stimulus, or in recovery [70]. Two EEG recordings were collected for 12 healthy participants, 14 days apart, and for three conditions. The first was a pre-cold pressor test condition as the baseline, the second was during the cold pressor test, and the last was during the recovery period following the conclusion of the CPT. The results showed that the theta and alpha frequencies were coupled with the gamma frequency during the CPT. This relation was significantly minimized during recovery and the theta power in the left frontocentral region rebounded significantly. The authors also observed that during the CPT there was a decrease in the theta, alpha, beta, and delta powers but an increase in gamma power. Also, there was a decrease in theta connectivity but a significant over-recovery [70].

Levitt et al. [71] investigated the findings of a preliminary rat study [87]—which detected theta-range cortical synchrony located in the somatosensory and prefrontal cortexes while assessing cortical synchrony in pain states—in a human population. The study consisted of 25 healthy subjects, 15 of whom underwent the cold pressor test and 10 of whom used only room-temperature water. EEG recordings and pain scores were recorded. The results showed that the stimuli resulted in increased synchrony in frontal EEG measurements but decreased synchrony in caudal EEG measurements. Connectivity also increased in the fronto-caudal and decreased in the fronto-parietal regions. This study suggested the need for further investigation of the correlation of synchrony to pain. For the subjects who experienced pain, the theta-range frontal EEG power increased significantly, suggesting that theta power is correlated to pain [71].

#### 3.2.3. Electrical Stimulation

DFNS QST, like most QST protocols, does not include an electrical stimulation component. However, this technique has been used to induce pain in experimental subjects. In addition to laser stimulation, Beck B. et al. [66] also utilized transcutaneous electrical stimulation to compare results between stimuli types. A DS5-isolated bipolar current stimulator delivered a 10 ms square-wave pulse to the inner left wrist. van den Berg et al. [73] investigated detection probability features and EEG data to assess the possibility of using them to detect and examine failed back surgery syndrome (FBSS). A total of 16 FBSS participants and 17 healthy control subjects received 450 intra-epidermal electrical stimuli on the backs of their hands, releasing a button when they detected the stimuli. The results showed a significant difference between all the healthy and FBSS subjects’ psychophysical features, larger detection thresholds, and lower detection rates. This suggested that the FBSS group experienced more difficulty in distinguishing the stimuli’s presence. Also, there was no significant difference between the groups regarding their EEG features. Finally, the differences between the groups were seen on the P2 vertex by the difference in amplitude of the second pulse [73].

#### 3.2.4. Mechanical Stimulation

The procedures for the mechanical stimulation portion of DFNS QST include pain sensitivity and thresholds. These are carried out by weighted pinpricks with forces between 8 and 512 mN. These assess Aδ-mediated hyperalgesia, hypoalgesia, or sensitivity to sharp object stimulation [86].

Ding et al. [78] used this component of the QST procedure, stimulating with 8–512 mN on the non-dominant forearm. First, a single stimulus was administered, followed by a 1 Hz series of 10 stimuli. Kenefati et al. [74] compared the behavioral and EEG responses of patients with chronic lower back pain (CLBP) to a mechanical stimulus in a pain area vs. a pain-free area. The same was assessed for healthy, pain-free individuals. EEG data were collected from 15 subjects with CLBP and 15 age- and gender-matched healthy controls. Both groups reported higher pain with the 256 mN stimulus at both locations. The CLBP group reported significantly higher pain in both locations compared to the control. The results showed that at the site of the pain, and only for the 256 mN stimulus, the CLBP group showed higher mean alpha and theta powers in the contralateral medial OFC. Also, at the pain-free site, only for the 256 mN stimulus, the CLBP group showed a higher mean theta power in the dorsolateral prefrontal cortex (dlPFC) and mean high gamma power in the anterior cingulate cortex (ACC). Overall, the study reported that the CLBP group had greater general pain sensitivity and gave insight into the frequency of associated mean powers [74].

#### 3.2.5. Other Stimulation Methods

Pressure pain stimulation is a component of DFNS QST; however, it is not a common method for experimental pain induction because it assesses deep pain sensitivity [86]. It is often used for pain threshold detection; thus, high pressures would be needed to elicit a strong pain response. Studies [60,72] utilized an algometer at the thenar and above the knee to collect three measurements of the minimum pain-eliciting pressure for each participant under normal conditions and again during a cold-pressor-conditioned pain procedure. Another study [81] conducted the same procedure but on the hand. For similar reasons, vibrational stimulation is not used to assess pain because of its intensity. Extremely high vibrations would be necessary to induce pain.

One common aspect in pain studies is incorporating nerve growth factor (NGF) to increase a subject’s sensitivity to pain. Chowdhury et al. [75] assessed the reliability of PAF and corticomotor excitability (CME) as potential cortical biomarkers for pain. This was achieved by observing the effect of sustained experimental pain on both factors. A total of 85 participants were injected with 0.2 mL of sterile recombinant human NGF in their right masseter muscle at the ends of day 0 and day 2 and observed for 30 days. Transmagnetic stimulation was utilized to measure corticomotor excitability. Electromyographic (EMG) recordings, transcranial magnetic stimulation responses, EEG recordings, and questionnaire data were collected on days 0, 2, and 5. The EEG data were acquired from 63 active electrodes using the Brain Products platform. The pipeline’s impact, recording length, frequency window, and peak identification method were assessed, since many factors, including the recording and derivation method, could influence the PAF. The results showed that the PAF was stable at each recording, suggesting its reliability. It also showed that the peak identification method had the most influence on the PAF, with excellent reliability through the center of gravity method. CME was assessed using 74 participants’ data; the results showed that 80 percent of the subjects exhibited either CME facilitation or depression with good stability, showing that it has good reliability as a pain biomarker [75].

Another study conducted by Furman et al. [76] investigated pain-free sensorimotor PAF as a potential predictive biomarker for pain sensitivity to musculoskeletal pain. The 31 healthy subjects were broken into two groups, one for each study. Study 1 consisted of five visits where EEG recordings were collected, and 0.2 mL of sterile recombinant human NGF was administered into the right extensor carpi radialis brevis muscle belly on days 0, 2, and 4. Similar procedures were followed in Study 2, consisting of three visits with NGF injections on days 0 and 2. For Study 2, transcranial magnetic stimulation was used for pain modulation after day 4 for five consecutive days. The data analysis was based on 17 subjects’ data for Study 1 and 10 for Study 2. The results showed a significantly high correlation between PAF and NGF pain sensitivity. They also showed that the pain scores after NGF administration were consistently greater for those with slower PAFs. Also, the PAFs were stable throughout the study period, regardless of pain. Finally, the results suggested that PAF shifts were not accurate markers of the presence of pain. Overall, the study concluded that PAF could be a marker of pain sensitivity in musculoskeletal pain [76].

## 4. Discussion

One of the review research questions pertains to the methodology of various pain studies and inquires about their different ways of inducing pain in participants. It leads us to understand how researchers accomplish similar tasks using different techniques and how they compare their results with the literature. To the best of our knowledge, no current systematic review highlights EEG responses in pain states while encompassing both chronic pain and experimentally induced pain methods, allowing for the comparison of signals from induced pain states and chronic pain. This section discusses and compares the biomarker findings in the studies. We gave special importance to frequency-band oscillations and powers since all the included studies report and assess them. Finally, we outline the limitations identified in the reviewed pain studies to highlight areas for improvement in future research.

### 4.1. Current Types of Experimentally Induced Pain

**Figure 4:**
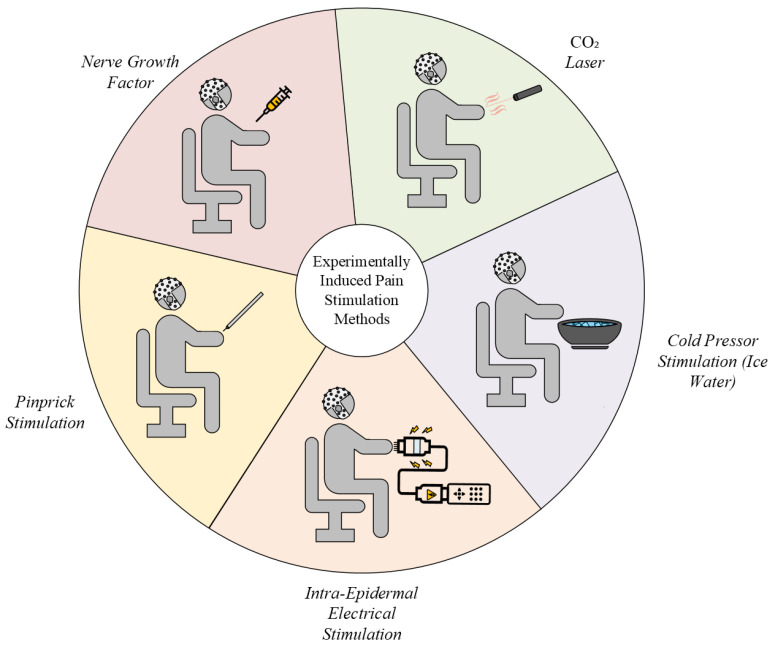
A visual summary of the different methods used to induce pain states experimentally: a CO_2_ laser is used as a heat stimulus, but similar effects can be achieved through the CHEPS. The participant’s hand is submerged in ice water for the CPT method. Intra-epidermal electrical stimulation consists of needles that penetrate the skin to deliver electrical stimuli. Mechanical stimulation often consists of using a weighted pinprick or a probe to apply pressure to the skin. Nerve growth factor is used to increase pain sensitivity around the area of injection by decreasing pain thresholds. This is why it may be paired with one of the other mentioned techniques.

The three overarching components of pain induction and measurement are a stimulus, a stimulation protocol, and data collection [88]. These components work together to create controlled pain scenarios, enabling researchers to study the neural, physiological, and psychological aspects of pain in a reproducible manner. The stimulus serves as a pain-inducing agent, directly activating pain-sensing neurons to elicit a physiological pain response. Common types of stimuli are thermal, electrical, mechanical, and chemical triggers. Incorporating techniques from DFNS QST, researchers use a multitude of stimulation procedures to experimentally induce pain, including thermal, electrical, mechanical, and chemical triggers. A summary of those methods is shown in Figure 4. The trigger excites the nerve-ending fibers that signal responses to the spinal cord and brain [89]. As a result of brain processes, changes in neural oscillations can be seen.

Thermal stimuli involve the controlled application of heat or cold to skin or tissues and are often used to study thresholds for pain perception, as in cases of burns or frostbite-like sensations. As heat is one of the most popular methods [62,64,65,66,67], the Contact Heat Evoked Potential Stimulator, or CHEPS, is a commonly used device. It uses a set of quick thermal pulses to activate A-delta and C fibers, which cause evoked potentials [90]. A-delta and C are nerve fibers responsible for two main components of pain: perception and intensity, respectively [89]. This makes them important parameters for pain research. One of the other methods of thermal stimulation is the use of a CO_2_ laser. Lasers emit a form of thermal radiation that, similarly to the CHEPS, activates the A-delta and C fibers, thus resulting in evoked potentials or laser-evoked potentials (LEPs) [91].

In the cold pressor test (CPT), participants are directed to submerge one of their hands or feet into ice water for about 1–3 min [60,62,68,69,70,71,72]. The water temperature varies greatly between studies, but the most common temperature for the CPT is 1 °C [92]. Variations regarding the equipment used in administering the CPT were present in the studies. Some studies used circulating water baths, pumping devices, or manual agitation, while others used still water [92]. Initially, the technique was designed to research variability in blood pressure [93]. In pain research, the application of the CPT translates to simulating chronic pain responses [94]. However, as the temperature drops become large and pose a risk of tissue damage, polymodal nociceptor fibers and cold-specific receptors activate and relay pain signals to the brain [95].

Electrical stimuli apply electrical currents to stimulate nerves artificially. This method is widely used because of its precision in controlling the stimulus intensity, duration, and localization. Applied electrical stimuli are used as a method of pain relief [59]. This can be achieved through using surface electrodes to decrease pain signals [96]. The method either activates A-beta fibers to hinder C fibers or activates A-delta fibers to stimulate encephalin. Encephalin is released in the spinal cord to hinder pain pathways [97]. Some cases use a dual-activation method to achieve better pain relief results, known as transcutaneous electrical nerve stimulation, a form of non-invasive neuromodulation [98]. More invasive methods are achieved with needles or implants that deliver the electrical signals. Although it is often used in clinical practice, clinical and research speculation exists on its efficacy [99]. Current research also utilizes similar methods to deliver stimuli to induce an experimental pain response from subjects in order to analyze neural activity [66,73,100]. Stimuli are typically delivered by square-wave pulses through surface electrodes [66,101] or intra-epidermal electrodes [73].

Mechanical stimuli include the application of pressure, vibrations, or sharp objects (e.g., pinpricks) to activate nociceptors. These methods are often used to replicate the sensations of blunt trauma or injury. Mechanical pain stimulation normally involves a device repeatedly applying force to a subject’s skin [74,102]. The applied forces range between 8 and 512 mN [86]. The same procedures can also be performed using a needle [103]. Mechanoreceptors are located throughout most layers of the skin and are responsible for facilitating the transformation of external stimuli into intracellular signals [104]. Mechanonociceptors facilitate the reception of the pain signals caused by noxious mechanical stimuli. These receptors are the main fibers of the peripheral nerve and transduce signals through sodium channels [105]. Similarly to how other pain receptors work, the free nerve endings are irritated, creating a chemical signal. The receptor converts this into an electrical signal and sends it to the spinal cord [106,107]. The electrical signal is then relayed to the brain, translating the signal and inducing a pain response [107].

Chemical irritants or compounds stimulate pain, mimicking conditions like inflammation or exposure to toxins, such as in capsaicin application for burning sensations [64]. Some studies used a chemical component to increase subject sensitivity to simulate chronic pain [75,76]. NGF is a peptide that contributes to the development of peripheral sensory and sympathetic neurons and has a role in adult hyperalgesia [108]. It has been utilized as a form of experimental pain induction using NGF injections. This leads to hypersensitivity and allodynia in the injection area [109]. It increases inflammatory responses, nociceptive receptor activity, nociceptive gene expression, and nerve terminal density [110]. Normally, this technique is paired with another form of pain stimulation. This helps to assess pain perception disparities between those with increased pain sensitivity and healthy controls. Additionally, it can be used to facilitate a period of chronic pain.

The stimulation protocol governs stimulus delivery, ensuring consistency and repeatability in pain experiments. Researchers design stimulation protocols to align with the goals of their studies, whether focusing on acute pain (short-term) or chronic pain models (long-term). The key factors are intensity, duration, frequency, and localization.

Data collection involves collecting data to measure physiological and neural responses to pain stimuli. These data provide insights into how pain is processed in the nervous system. Electrophysiological measurements such as electroencephalography (EEG) or magnetoencephalography (MEG) capture changes in neural oscillations, revealing brain activity associated with pain processing. Functional imaging techniques such as functional MRI (fMRI) or positron emission tomography (PET) track pain-related brain activation by measuring changes in blood flow or metabolic activity. Behavioral responses—collecting subjective reports of pain intensity, tolerance, and location using pain scales like the VAS or numerical rating scales—and autonomic responses—measuring physiological changes such as heart rate, skin conductance, or pupil dilation—are also common forms of data collection.

### 4.2. Pain Biomarkers and the Effects of Pain on the Electrical Activity of the Brain

Functional connectivity (FC) measures use EEG data to assess the relationships or interactions between different brain regions, often by analyzing the synchronization or coordination of electrical activity [111,112]. They provide much information on brain activation, communication, and structural connections [113]. FC methods can be classified into linear, non-linear, and network-based approaches, each providing insights into neural communication and functional networks. The coherence model of the linear approach is commonly used in pain studies. It assumes a linear relationship between signals from different EEG channels and measures the consistency of the phase relationships between two signals at specific frequencies. It assesses the synchronization between regions in pain studies, but cannot distinguish between direct and indirect interactions. Phase locking value (PLV) and mutual information (MI) are non-linear models capturing complex and non-linear relationships in pain perception and chronic pain using EEGs. PLV focuses only on the phase relationships and not amplitude dynamics, quantifying the consistency of phase differences between two signals, irrespective of amplitude. Computationally intense MI models measure the shared information between two signals, capturing linear and non-linear dependencies. Network-based methods use graph theory to analyze functional connectivity as a network of brain regions and connections. Global efficiency is one of the models that uses graph theory to quantify information transfer efficiency across the entire network in conditions like chronic pain. However, it can also find local connectivity patterns. Similarly, local efficiency models assess changes in specific brain regions, such as the prefrontal cortex or somatosensory areas, and consequently require a high spatial resolution. Network-level dynamics can be captured by combining global and local efficiency models, which requires high-density EEG measurements. Cross-frequency coupling (CFC) is another approach to measuring FC, requiring advanced mathematical techniques and careful interpretation. With multi-frequency insights, CFC analyzes how slow and fast oscillations interact in pain-related processes. The technique requires the extraction of phase and amplitude from the signals, followed by correlation assessments between the features, and finally statistical significance calculation [114]. All these methods measure FC in the brain with a specific focus. It is important to understand how FC is affected during pain stimulation. This gives insight into the reaction times of neural pain processing. Our review observes that comparing studies on a single aspect is difficult, as each finding was based on different conditions, although many studies investigated pain and different brain regions. However, there does exist some degree of correlation between different neural locations. Some studies reported decreased FC in theta bands during pain stimulation [59,60]. Also, FC increased in areas such as the fronto-caudal region [71], brainstem [61], and cerebellum [61]. An increase in alpha FC was seen with the alleviation of pain [59]. The literature suggests that FCs might be directionally dependent; one of the studies showed that the FC was higher from the pregenual cingulate to the somatosensory cortex but lower in the reversed path [59]. In addition to variations in experimental setup and analysis, some other limitations to generalizing such results are that some studies have shown that FC may be individualized to some degree. They reported that individual anatomical variations in brain regions could highly influence FC mappings [115]. The subjective nature of pain also affects functional connectivity. Pain’s sensory–discriminative and affective–motivational components and subcomponents create a complex perceptional system. Thus, FC would reflect the processing of the pain perception subcomponents, which would take place in different brain regions. Many fMRI studies have analyzed how pain perception relates to FC [39,116] and how abnormal pain perception is reflected in altered FCs [117,118], supporting the FC variations seen in EEG pain studies.

Frequency oscillations are some of the most commonly assessed brain features. Studies have shown that band frequencies tend to differ in the presence of different kinds of pain and duration. Some bands have been seen to behave differently than those at higher or lower frequencies. Alpha bands range from 8 to 12 Hz, and studies have shown that pain is associated with slower alpha oscillations [76]. To support this finding, pain alleviation increased these oscillations, which is mostly consistent with other studies investigating pain and EEG frequencies [119,120]. The alpha band is affected by the intensity and duration of pain, suggesting that it has the potential to be a pain biomarker. The reports for the theta band suggest that observed oscillations may be useful in pain state and type discrimination along with long-term monitoring. They can be employed with alpha band observation to better define a pain experience. The literature states opposing correlations between observed oscillations in the presence of chronic pain and neuropathic pain [60]. Gamma oscillations may also be used for pain discrimination, as they show different behaviors in response to different types of stimuli. Studies have been performed to analyze the role of the gamma band in pain processing. It has been found that pain perception is highly linked to gamma-band activity [121]. Gamma-band activity plays a large role in pain processing. Li et al. [122] showed that pain results in higher gamma-band activity in the frontal–central region of the brain and that gamma-band amplitudes positively correlate with pain intensity. Other studies have reported similar relationships and behaviors between gamma-band activity and pain [123,124,125]. It has also been seen to represent different components of pain [126].

Frequency power is used to quantify the degree of oscillation present in the signal [127]. Using Fourier transforms and similar techniques, band power can be extracted from the total signal power [128] and used to analyze which frequency ranges influence the overall signal of a specific pain state. It was reported that stimulated pain intensity and chronic pain severity increase beta power [60,62]. However, based on CPT results, the beta power can also decrease with pain [70]. For the theta band, in some cases, the intensity of the stimuli and chronic pain severity lead to decreased theta power in the presence of pain [60]. In other cases, an increase in theta power is observed [55,71,74]. These variations could stem from differences in experimental design, data analysis methods, or sample size. For the alpha band, its power was correlated to motor-EP and decreased in the presence of pain, as it is consistent with the decrease in alpha oscillations [63,64,65,76]. Delta power was reported to increase in pain states and decrease with pain modulation [72]. However, some CPT studies reported decreases, which resulted in increased gamma power [70]. Frequency power can be used to determine frequency-band coupling, which refers to the synchronization and relationship between different bands [129,130]. For this reason, frequency coupling is also affected by pain states. With the presence of pain, the theta and alpha bands couple with gamma bands. In instances of pain alleviation or recovery, this relationship decreases. The reviewed studies agree that band power and band coupling are potential biomarkers for pain because the amount of activity in certain bands changes depending on the presence or severity of pain.

### 4.3. Major Findings for Chronic and Experimentally Induced Pain from EEGs

Preparing a logical framework is challenging because experimentally induced pain models, which are transient and lack the prolonged neural plasticity associated with chronic conditions, do not fully capture the emotional and psychological dimensions of chronic pain. Instead, we listed the major findings in PAF, prefrontal control, emotional networks, and the DMN for the pain categories in Table 3.

Heitman et al. [61] indicated a positive relationship between changes in pain-related disability and local degree in the beta band in the cerebellum, though this was observed in only one clinical measure. Although the functional significance is unclear, because the reliability of detecting cerebellar signals using EEGs remains uncertain, further analyses showed that decreases in pain intensity and disability following IMPT were associated with increases in gEff in the theta band. Cross-sectional studies comparing local and global brain network measures in chronic pain patients and healthy participants have yielded inconsistent results [61]. Previous cross-sectional studies have observed decreased global connectivity at theta and gamma frequencies. However, the present longitudinal findings suggest a positive relationship between improvements in chronic pain and increases in global network efficiency at theta frequencies.

There was no evidence of coupling between the PAF and corticomotor excitability in experimentally induced pain using EEGs [75]. An MRI study [131] provided evidence of functional coupling in chronic pain across multiple frequency bands—theta, alpha, beta, gamma—between the salience network and three key networks: the ascending nociceptive pathway, the descending anti-nociceptive pathway, and the default mode network (DMN), which is active when no pain stimuli are perceived and inactivate when stimuli are presented [132]. As the posterior cingulate gyrus is the central hub of the DMN, studies reported increased connectivity in the gyrus in chronic pain. Table 3 summarizes the major findings of neural activity in chronic and induced pain regarding PAF, prefrontal control, emotional networks, and DMN features.

#### 4.3.1. Chronic Pain Band Oscillations and Power Markers

Higher pain intensity (measured by a VAS) was associated with lower alpha and beta powers within the SCI pain group in the Simis et al. study [81], further reinforcing that reduced inhibitory cortical activity is linked to greater pain perception. Uygur-Kucukseyment et al. [82] reported a similar marker—lower alpha and beta powers over the central region in resting EEGs being associated with higher pain levels—for FM patients. In contrast, SCI patients without pain exhibited stronger alpha and beta oscillations, suggesting a protective mechanism against pain development. Although the increase in theta power was not statistically significant, there was a trend toward lower EEG frequency shifts, marked by a reduction in peak theta–alpha frequency (PTAF), in the pain group. The presence of thalamocortical dysrhythmia, characterized by an increase in theta-band power and a decrease in alpha power, was observed in the pain group. This dysrhythmia is recognized as a key feature of neuropathic pain and suggests impaired cortical inhibitory mechanisms, leading to an enhanced pain experience. In contrast, SCI patients without pain maintained more stable alpha and beta oscillations, suggesting a better-preserved cortical inhibitory system, possibly contributing to pain suppression [81]. A significant negative correlation between beta power and pain levels may be a result of patients with higher pain levels exhibiting lower beta band power in the central and frontal regions. This reduction in beta power reflects a disrupted balance between inhibitory and excitatory systems, contributing to hyperexcitability and chronic pain. SCI patients without pain had relatively higher beta powers, possibly indicating a compensatory mechanism that helps to regulate pain processing [81].

Contradictory to Simis et al.’s report [81], Barbosa et al. [77] reported increased alpha oscillations in the central region, which correlates with higher reported pain intensities for SCI patients. The SCI patients without pain did not show the same level of increased alpha activity, suggesting better natural pain regulation and inhibition mechanisms in Barbosa’s study. On the other hand, higher theta activity in the frontal and parietal regions was linked to increased pain intensity in SCI patients with pain. Increased delta oscillations were observed in the resting-state EEG, suggesting a potential compensatory role where the brain attempts to mitigate pain perception by reducing the focused attention given to pain-related stimuli. Beta oscillations were not found to be significant as a marker. Reference [80] supported the idea that theta power is higher in sickle cell disease (SCD) patients compared to its healthy control group, but also reported findings that the patient group tended to have more power in the low-frequency bands (delta, theta, and alpha) and less power in the high-frequency bands (beta1, beta2, and gamma).

A distinct EEG signature was reported between patients with FM/chronic pain and those without pain [79]. A negative correlation between beta oscillations and pain intensity was linked to the higher beta powers associated with lower pain levels. In contrast, patients with more severe and long-term pain showed increased low-frequency power (delta and theta bands), suggesting a disrupted cortical state. Beta oscillations are associated with inhibitory GABAergic activity, which plays a role in pain modulation. Lower beta power in patients with pain may indicate a disruption in cortical inhibitory mechanisms, leading to increased pain experiences. Higher beta oscillations in pain-free individuals suggest a better-modulated pain inhibitory system and greater cortical stability. Pain-free individuals displayed more-stable EEG patterns, particularly in the beta range, reinforcing the role of beta oscillations in pain inhibition. FM duration was associated with increased power in the low-frequency bands (delta and theta) and beta power, suggesting long-term adaptations in cortical processing. These findings align with Case’s [80].

Most of the studies were conducted with adolescents and adults. One of the rare works in the literature concerning pediatric patients was Ocay et al.’s [62] study on the EEG markers of children and adolescents with chronic musculoskeletal (MSK) pain. A significant age-related decrease in resting global theta power was only observed in the CP group, and no other age-related associations were observed. Patients with pain (CPs) exhibited altered EEG patterns compared to the healthy controls, particularly showing increased resting delta and beta powers, suppressed delta/theta powers during tonic heat pain, and an increased peak beta frequency during cold pain testing (CPT). Pain alters both baseline and stimulus-induced brain activity, likely reflecting cortical reorganization and disrupted pain modulation mechanisms in CPs.

Several studies [79,81,82] consistently reported lower alpha and beta powers in patients with pain, particularly in SCI and FM populations, correlating with higher pain intensity. This suggests that reduced inhibitory cortical activity, likely mediated by GABAergic dysfunction, is a key feature of chronic pain. In contrast, pain-free individuals exhibit stronger alpha and beta oscillations, which may serve as a protective mechanism against pain development by enhancing top-down inhibitory control. While Simis et al. [81] and Uygur-Kucukseyment et al. [82] found lower alpha powers in pain patients, Barbosa et al. reported higher alpha powers in the central region, correlating with increased pain intensity in SCI patients. This discrepancy may be due to differences in pain chronicity, methodology, or patient subtypes. Several studies [79,81] linked lower beta power to higher pain intensity, supporting the role of beta oscillations in pain inhibition. However, Barbosa et al. [77] did not find beta oscillations to be a significant marker of pain, suggesting that beta power alterations might be more relevant to specific pain conditions or stages of chronic pain progression. Some studies [77,80] reported increased delta oscillations in patients with pain, suggesting a compensatory mechanism where the brain reduces its focus on pain stimuli. In contrast, other studies emphasized increased delta power as a sign of cortical dysfunction rather than a protective mechanism. Delta oscillations’ dual nature might depend on individual pain perception and underlying neurophysiological adaptations.

These findings indicate that EEG alterations in patients with pain are complex, involving multiple frequency bands and compensatory mechanisms. While alpha, beta, and theta dysregulation are commonly observed in chronic pain populations, inconsistencies in the findings suggest that pain-related neural changes may be condition-specific, influenced by pain chronicity, or experiment-dependent. Future research should aim to standardize EEG methodologies, explore longitudinal pain adaptations, and investigate individualized pain signatures for more precise clinical applications.

#### 4.3.2. Induced Pain Band Oscillations and Power Markers

Furman et al. [76] studied peak alpha frequency (PAF) stability in patients with induced pain and healthy controls. In patients with pain, sensorimotor PAF remained highly stable over a 14-day period, despite the patients experiencing ongoing NGF-induced pain. In contrast, healthy controls (pain-free individuals) exhibited naturally occurring variations in PAF, suggesting that PAF fluctuations are not necessarily linked to pain persistence. The presence of pain did not lead to a systematic slowing of PAF, indicating that PAF changes may not be a direct consequence of prolonged pain experiences. The study believed that its findings propose that a slow PAF in pain-free individuals may indicate an increased risk of developing chronic pain. Healthy controls with slower pain-free PAFs (~10 Hz) might be more susceptible to chronic pain following injury or pain-inducing stimuli. It suggested that PAF slowing is not a marker of active disease but rather a pre-existing neural characteristic associated with heightened pain sensitivity. Although PAF is not a consequence of pain persistence, it may be a predisposing factor for chronic pain. If so, healthy controls with slower PAFs may be at a higher risk of developing pain conditions than those with naturally faster PAFs. PAF-based screening in pain-free individuals could revolutionize early pain prevention strategies, making it a valuable biomarker for identifying individuals at risk before chronic pain develops. On the other hand, Chowdhury et al. [75] used the same induced pain method (NGF) and reported that peak alpha frequency (PAF) remains stable even in the presence of experimentally induced pain, suggesting that pain does not significantly alter the fundamental oscillatory properties of alpha activity. This contrasts with studies on chronic pain patients, where altered PAFs have been observed, indicating possible differences between induced pain and chronic pain states. Also, in the healthy controls, PAF remained consistent across sessions, reinforcing the idea that it is a stable individual trait, minimally affected by temporary discomfort. The study in [64] demonstrated that pain-free PAF is a reliable predictor of prolonged pain sensitivity. It suggests that monitoring PAF in clinical settings may help in personalized pain management strategies, allowing for targeted treatments based on individual neurophysiological pain sensitivity profiles.

Levitt et al. [71] used a cold pressor test to induce pain and reported increased theta power in the frontal cortex during pain, which is consistent with previous human studies, highlighting theta oscillations as a potential biomarker of pain. Reduced alpha power has also been suggested to predict pain intensity, aligning with prior research on tonic heat and cold pressor pain models. Healthy controls did not show these theta and alpha shifts, further reinforcing the role of these rhythms in pain perception. Painful stimulation led to increased frontal theta power (6–7 Hz) at the Fz electrode and decreased caudal EEG power (3–30 Hz) at the O1 electrode, reflecting a pain-induced modulation of low-frequency oscillations. Healthy controls without pain showed no significant EEG power changes, suggesting that pain uniquely alters oscillatory activity in cortical regions. Patients with pain exhibited enhanced fronto-caudal connectivity and reduced fronto-parietal connectivity, suggesting a reorganization of cortical networks in response to pain. In healthy subjects, fronto-parietal connectivity remained stable; therefore, this change was pain-dependent rather than a general neural fluctuation. Another cold pressor test [70] reported a significant reduction in theta and alpha power at the central electrode sites, bilaterally. A theta power decrease was observed at the C3, P3, Cz, C4, and Fz electrodes (all *p* < 0.05), with the strongest effect at C3 (tmin = −4.9). Alpha power decreases were primarily observed at the C3 and C4 electrodes (both *p* < 0.05), with the greatest reduction at C3 (tmin = −2.6). The high gamma power significantly increased in the frontal regions, particularly at the Fz and F4 electrodes (both *p* < 0.05). The most significant gamma power increase was observed at F4 (tmax = 3.1). This increase suggests enhanced cognitive and attentional engagement during pain processing, as gamma oscillations are often linked to pain perception and top-down modulation.

The reported biomarkers for induced pain are consistent, while chronic pain biomarkers show inconsistencies. The differences may arise from fundamental distinctions in pain mechanisms, experimental control, and neurophysiological adaptations over time. Induced pain studies use controlled experimental paradigms, such as thermal, electrical, mechanical, or pressure stimuli, applied to healthy participants or patients under highly regulated conditions. Consistency in stimulus intensity, duration, and application site ensures that the recorded EEG or neuroimaging responses reflect similar neurophysiological processes across studies. In contrast, chronic pain varies across individuals in intensity, duration, and underlying pathology, making the biomarker findings less uniform.

Induced pain activates well-defined, transient neural circuits involving the somatosensory cortex, anterior cingulate cortex (ACC), insula, and thalamus, producing short-term, consistent neural responses. Chronic pain leads to long-term maladaptive neuroplasticity, involving cortical reorganization, altered thalamocortical rhythms, and impaired descending pain modulation, which varies across patients based on pain duration, severity, and comorbidities. Chronic pain also interacts with affective and cognitive processes (e.g., anxiety, depression, catastrophizing), further influencing the variability of biomarkers.

### 4.4. Applications

Once pain neuro markers have been identified, they can be applied in real-world interventions to enhance pain assessment and management. Most of the studies reviewed are conceptual or laboratory-based, and their findings primarily suggest therapy and pain intervention as potential future applications. Carina Graversen et al. [133] suggested that pain biomarkers could be utilized in basic and clinical research to investigate nervous system changes in response to pain. Additionally, they may serve as tools in clinical trial settings, facilitating targeted subject enrollment for pharmaceutical testing.

A limited number of commercial products have been developed to assess and manage pain by leveraging neurophysiological markers. An AI-powered medical device company, PainQx, has developed a next-generation approach to objectively measuring chronic pain in humans. The PainQx platform is an AI-powered cloud-based solution that operates on EEG data collected from a patient, processing the data through proprietary algorithms and classifying the patient’s pain state for the clinician at the point of care. The system collects neural activity data from the patient’s brain using a mobile and low-cost EEG device. It automatically transmits its data to the cloud, where PainQx’s proprietary algorithms process and decode the information. The processed data are analyzed to classify the patient’s pain state into the following categories: no pain, mild/moderate pain, or severe pain. This classification correlates with the patient’s self-reported Numeric Rating Scale (NRS) pain score. The resulting pain classification is provided to the clinician at the point of care, offering an objective assessment to support treatment decisions and monitor treatment efficacy. The PainQx platform aims to improve patient outcomes, reduce the reliance on subjective pain reporting, and address challenges in pain management, such as under- or over-treatment [134].

Neurostimulation devices have been developed to manage chronic pain by targeting neural pathways, though they do not directly employ pain neuro markers. Medtronic’s Intellis™ Spinal Cord Stimulation (SCS) System, a rechargeable neurostimulation system, aids in managing certain types of chronic, intractable pain by delivering mild electrical pulses to the spinal cord, thereby altering pain signals before they reach the brain [135]. Abbott’s Eterna™ SCS System is another neurostimulation device, featuring BurstDR™ stimulation designed to mimic natural patterns in the brain. This system provides relief from pain and its associated impacts, enabling patients to resume daily activities [136]. While these devices modulate neural activity to alleviate pain, they do not incorporate specific neuro markers for pain assessment or personalized treatment.

The development and validation of such biomarkers remain under investigation due to biological variability, methodological limitations, and the subjective nature of pain perception. These complexities must be addressed in order to advance pain diagnosis and enable the development of personalized therapeutic interventions in the future.

### 4.5. Limitations of Pain Studies and Future Directions

One of the most commonly reported limitations in analyzing these pain research studies was a small and non-diverse sample size [59,68,69,70,74,77,78,80,81,82]. A small sample size can decrease the reliability of results. Concluding such results may prove to be invalid due to increased mean squared error and standard deviation, which can lead to low statistical power, increasing the chances that any statistically significant results do not reflect fact. Unfortunately, the average sample size in neuroscience has not increased over the years, resulting in the lower statistical power of results [137]. By increasing the number of subjects, study results can generalize the impacts and biomarkers of pain in the brain.

Another common limitation is the lack or insufficient nature of control groups [60,61,65,72,75,79,81,82]. When considering a treatment factor among subjects, it is important to include a control group. This set of participants should be as similar to the treatment group as possible, with the only difference being the lack of the treatment factor. It highlights the true differences in the response variables and increases signal-to-noise ratios [138]. In pain research, the lack of a control group or sham stimulus can lead to flawed analysis, as failing to establish a baseline for comparison complicates the interpretation of the results and diminishes the reliability of conclusions. Controlling variables, such as time intervals, by holding them as a constant [66,67,68,74] may also pose a drawback.

Participant factors are an inherent aspect of human studies. While some variables, like age, gender, and other demographics, can often be controlled, others may not always be manageable [68]. Factors such as variations in biophysical parameters [62], medications [80,81], comorbidities [60,61,63], and behavioral differences [67] are challenging or even impossible to regulate. Common comorbidities of chronic pain or chronic widespread pain include diabetes, irritable bowel syndrome, neuropsychiatric disorders, joint pain, peripheral nerve damage, headache, and chronic fatigue syndrome [60,61,139]. In pain research, where sample sizes are typically small, controlling these factors can further reduce group sizes, increasing the risk of errors in data analysis and interpretation. Additional influences include subjective differences, such as variability in baseline conditions [62] or differences in stimulus severity [68,74,78].

Pain studies often face the limitation that findings from one specific pain or stimulus type cannot be generalized to others [61,62,66,67,70,74]. For instance, concluding that the characteristics of chronic lower back pain apply to all chronic pain patients without proper comparative studies would be inaccurate. Similarly, including all chronic pain types in a single study is impractical due to the challenges of controlling for variability. Generalizing results to diverse populations or encompassing multiple subpopulations in one study is usually not feasible. Thus, generalized conclusions can only be made by reviewing the literature in order to compare the findings of many studies.

Another limitation lies in the QST design, which evaluates pain perception by systematically applying controlled sensory stimuli and measuring study participants’ responses. The QST significantly impacts the study findings. Unfortunately, the studies in the literature often ignore or fail to present their QST design in detail, especially the application of stimuli. Developing standardized experimental setups is an urgent issue that needs to be addressed.

There are also reported statistical limitations in preprocessing, postprocessing, and analysis methods. Studies mentioned that data correction methods [59] or the lack thereof [68,72] may influence their analysis power or results.

Despite the limitations of the studies and the systems employed, EEG pain biomarkers are still a paramount research investment. There is an increasing demand for non-invasive, wearable, affordable, and practical pain assessment and monitoring systems that can be used in home settings and clinics. It is an emerging field that has started to gather attention in recent years. EEGs are currently the only potential technology that can meet such criteria. With advancing hardware and software technologies, EEG signal quality—its temporal and spatial resolution—is rapidly improving. EEGs are a more accessible, less costly approach to neuroimaging with temporal advantages, and they possess the qualities needed to address this demand [140]. This review identifies the EEG biomarkers representing chronic and induced pain in humans and highlights the differences in them between pain states. With further development, these biomarkers can be refined into diagnostic characteristics for different types of pain for translational applications.

Possible future work to address participant factors in pain research may include using larger and more diverse cohorts, which can be achieved through conducting multicenter studies or establishing collaborative networks to recruit diverse participants across ages, genders, ethnicities, and medical backgrounds. This will enhance the generalizability of findings and reduce the impact of outliers or confounding variables. Standardized methodologies are also needed to develop universally accepted protocols for assessing and controlling participant factors. They may use validated tools to consistently measure baseline conditions, biophysical parameters, and behavioral differences across studies while minimizing variability due to inconsistent data collection and improving comparability between studies.

Because of the challenges of participant recruitment, most studies have small sample sizes. Unfortunately, this affects the robustness and reliability of the studies’ findings. Developing advanced statistical methods or employing machine learning algorithms to improve the handling of confounding variables in small-sample studies may increase the robustness and reliability of their findings despite their small sample sizes. Longitudinal study designs are also needed to reduce the impact of transient or situational factors and provide insights into the dynamic nature of pain.

The EEG data in pain studies are collected in laboratory settings for a relatively short period instead of capturing continuous, real-world data to understand biophysical and behavioral variations better. Using wearable and real-time monitoring technology is an effective way of collecting chronic pain data in naturalistic settings. Wearable EEG technology and signal acquisition quality have constantly improved over the years. It is now possible to collect longitudinal data to account for day-to-day variability in participants. This will provide richer datasets, reduce reliance on single-point measurements, and allow the development of personalized pain profiles to account for individual variability. It will also enhance the precision of pain research and intervention strategies.

Comparison between studies is challenging since, typically, studies build their own datasets to analyze pain. Creating repositories of participant data for meta-analysis and model validation is critical in pain studies. Non-profit associations for pain studies should establish universally accepted guidelines for QST and brain studies to improve reproducibility and develop open-access platforms with well-defined data collection guidelines where researchers can upload their datasets, including detailed information about demographics, biophysical parameters, and pain-related metrics. Such comprehensive datasets will support large-scale analyses and reduce the need to replicate small-sample studies.

By implementing these strategies, future research can better address participant-, methodology-, and dataset-related variability, improving pain studies’ reliability, generalizability, and clinical relevance.

## 5. Summary and Conclusions

The assessment of electrical signals in the brain provides a better understanding of neural functions and processing. To investigate pain and derive its biomarkers, EEG signals of subjects in different pain conditions and intensities have been assessed to determine variables that influence and are affected by pain. In some studies, acute pain is experimentally induced using thermal, electrical, or mechanical stimuli. Other times, the subjects are present for the study of pre-existing pain, such as chronic pain from injury or surgery. However, such studies have many limitations, as the number of subjects is generally quite limited and there are methodological inconsistencies from study to study. This makes comparing and generalizing pain biomarkers difficult based on study findings. The techniques used by each study have some differences; this means that the results of each study are concluded based on different methods. Although there may be differences, there is a general trend in most of the findings: the studies suggest that the presence and level of pain affect neural frequency oscillations, functional connectivity, and frequency power.

To be able to conclude with more concrete results, future studies should reproduce current studies in similar but larger populations. Additionally, future studies should include variations in participant characteristics to analyze the differences or lack thereof in results between populations. Also, creating a standard for EEG recording techniques and pain stimulation methods would enable the robust comparison of study findings.

There is a lack of extensive research studying different factors in pain and brain interaction. Various factors affect pain, such as physical condition, age, gender, and emotional and mental states. Further research is needed to introduce these factors and investigate their influence on the brain. This will shed light on individual factors impacting pain perception in the brain and intervention strategies.

## Figures and Tables

**Table 1:** Summary of participants, study equipment, and stimulation methods.

Author (Year)	Participant Profile [# of Participants in Analysis]	EEG Equipment	EEG Acquisition Method [Sampling Frequency]	Additional Data Acquisition	Stimulation Method	Pain Scoring Method
Barbosa SP et al. (2024) [77]	Chronic neuropathic pain patients (>3 months), long–term pharmacological treatment (≥4 weeks) Male and Female Age: 18+ [36 participants]	EASYCAP (EASYCAP GmbH, Herrsching, Germany) 24 channels 10–20 system	Cross–sectional Study, [83] Zolezzi DM, et al. (2023) Resting state 10 min (Eyes open for 5 min, Eyes closed for 5 min) [250 Hz]	N/A or not mentioned in study	N/A or not mentioned in study	Brief Pain Inventory painDETECT [83]
Beck B et al. (2019) [66]	Healthy Right–handed Non–Sensitive Dark Skin Male and Female Age: 19–37 [57 participants]	ActiveTwo system (Biosemi, Amsterdam, The Netherlands) 64 channels	2 sessions EEG recording for each block [1024 Hz]	5 electrode Electrooculogram (artifact removal) Skin Temperature Measurement	CO_2_ laser (LSD, SIFEC, Ferrières, Belgium): wavelength (10.6 μm), spot size (6 mm diameter), 100 ms Transcutaneous electrical stimulation (Digitimer, Welwyn Garden City, UK): DS5 isolated bipolar current stimulator, square wave pulse, 10 ms. Recorded using Labview 2012 (National Instruments, Austin, TX, USA)	N/A or not mentioned in study
Camargo L et al. (2024) [79]	Chronic pain (diagnosis of fibromyalgia pain, ACR 2010 criteria, pain level of 4 (VAS) > 6 months) Age: 16–65 [EEG analysis: 73 participants]	EGI system (EGI, Eugene, OR, USA) 64 channels	Resting state 5 min (eyes open) motor–imagery–observational task block [1000 Hz]	CPM	Test stimulus: Peltier thermode (Medoc Advanced Medical Systems, Ramat Yishai, Israel): 30 s at pain–60 temperature, right forearm Conditioned stimulus: cold water, 10 to 12 °C, left hand, 30 s	VAS (pain) Brief pain inventory (pain symptoms) Patient–Reported Outcomes Measurement Information System (pain interference) numerical pain scale
Chouchou F, Perchet C, Garcia-Larrea L. (2021) [69]	Healthy Male and Female Age: 18+ [14 participants]	EEG Cap (Waveguard Cap, ANT) 128 channels 10–10 system	Baseline: 20 s, eyes open Grimace: 10 s. Rest: 90 s. Baseline Cold Pressure Test: 120 s. [1024 Hz]	N/A or not mentioned in study	CPT: 10 °C, 120 s. Participants were able to remove their hand if pain became unbearable Second condition: mimicking facial responses to pain	VAS
Chowdhury NS et al. (2023) [75]	Healthy Male and Female Age: 18–43 [PAF Analysis: 75 participants] [CME Analysis: 74 participants]	Brain Products platform (BrainVision Recorder, Vers. 1.22.0101 with actiCHamp Plus, Brain Products GmbH, Gliching, Germany) 63 Channels 10–10 System	Eyes closed 5 min recording	EMG	NGF: Right masseter muscle TMS for CME	11–point Numerical Rating Scale (NRS): 0 = “no pain”, 10 = “worst imaginable pain”
De Vries M et al. (2013) [63]	Chronic Pain Group (pancreatitis patients with persistent abdominal pain) HC (non–chronic pain patients) Male and Female Age: 18+ [Chronic Pain: 16 participants] [HC: 16 participants]	Quickcap (NuAmps, Compumedics Neuroscan, Singen, Germany) 26 channels 10–20 system	Eyes open and eyes closed, 2 min each Eyes closed for data analysis, [500 Hz]	EMG/EOG	N/A or not mentioned in study	N/A or not mentioned in study
Ding K et al. (2024) [78]	Chronic pain group: clinical diagnosis of central sensitization HC: healthy, non–chronic pain patients [Chronic: 6 participants] [HC: 8 participants]	WaveguardTM caps (ANT Neuro, Netherlands) *OR* SynAmps2 system (Compumedics Neuroscan, NC, USA) 64 channels	Resting state: 4 min (2 min eyes open, 2 min eyes closed) Recording continued through the QST procedure [500 Hz]	QST	Thermode (TSA2, Medoc, Ramat Yishay, Israel): 3 trails, 45 °C, volar of non–dominant forearm, 10 pulses (0.5 s each, 2.5 s interpulse interval) Weighted pinprick (MRC Systems, Heidelberg, Germany): 8–512 mN, volar of non–dominant forearm, single stimulus followed by 10–stimulus train (1 Hz)	Verbal numerical scale rating: 0–100 (0 = “no pain”, 10 = “worst imaginable pain”)
Furman AJ, Thapa T et al. (2019) [76]	Healthy Right–handed Male and Female Age: 18+ [Study 1: 17 participants] [Study 2: 10 participants]	Neuroscan SynAmps 2 digital signal–processing system and electrode cap (Compumedics Neuroscan, NC, USA) 19 channels	Resting state Eyes closed 3 min [1000 Hz]	TMS mapping EOG for artifacts	NGF injection: 0.2 mL (1–mL syringe, 27-gauge needle)	11–point numerical rating scale (0 = “no pain”, 10 = “worst imaginable pain”)
Furman AJ, Prokhorenko M et al. (2020) [64]	Pain–free Neurotypical Male and Female Age: 21–42 [Visit 1: 57 participants] [Visit 2: 43 participants]	EEG Cap (BrainVision actiCap system, Brain Products GmbH, Munich, Germany) 63 channels 10–20 system	2 visits (3 wks. apart) Eyes closed: 2 min Room temp thermode (5 min), phasic pain (5 min), recording pain intensity Ice: (5 min), eyes closed 40 °C thermode over capsaicin area (5 min) eyes closed, pain intensity recorded [500 Hz]	QST	Thermal Contact Heat Stimulator: 27 mm diameter Medoc Pathway CHEPS Peltier device (Medoc Advanced Medical Systems Ltd., Raman Yishai, Israel) Phasic heat Pain and Capsaicin Heat Pain: capsaicin paste (Professional Arts Pharmacy, Baltimore, MD) Icepack application	Manual Analog Scale: Rating from 0 to 100 (0 = “no pain”, 100 = “worst imaginable pain”)
Heitmann H et al. (2022) [61]	Chronic Pain (clinical diagnosis) Male and Female, Age: 18+ [41 participants]	“EEG Cap (EasyCap, Herrsching, Germany) & BrainAmp MR plus amplifier (Brain Products, Munich, Germany) 64 channels 10–20 system	Two sessions (5 min each) Recording A: eyes open Recording B: eyes closed [1000 Hz]	Interdisciplinary multimodal pain therapy (Center for Interdisciplinary Pain Medicine of the Technical University of Munich)	IMPT program BrainVision Analyzer software v 2.1 (Brain Products, Gilching Germany)	NRS: 0 = “no pain”, 10 = “maximum pain”
Kenefati G et al. (2023) [74]	Chronic pain group (CLBP) (pain > 6 mo, w/score > 4 on an 11–point pain scale) HC (no chronic pain) Age: 18–75 [CLBP Analysis: 15 participants] [Control Analysis: 15 participants]	High density EEG cap (Quick–Cap Neo Net, Compumedics Neuroscan, Charlotte, NC, USA) 64 Channels	Blindfolded w/mask Two sessions (5 min each) Recording A: eyes open Recording B: eyes closed [1000 Hz]	EOG Time–Frequency Representations	Weighted Mechanical Pinprick Stimulators (MRC Systems GmbH, Heidelberg, Germany): force of 32 mN and 256 mN, ~10 trials per force, interstimulus (10 s)	NRS: 0 = “no pain”, 10 = “worst imaginable pain”
Levitt J et al. (2017) [71]	Healthy Male and Female Age: 18+ [Ice water group: 12 participants] [Room temperature water group: 8]	MicroEEG and Statnet System (BioSignal Group, Acton, MA) portable EEG platform 16 channels 10–20 system	Resting state Eyes open (30 s) Eyes closed (60 s) Eyes open (30 s) Water/Ice (20 s): report pain after 5 and 15 s Eyes open (90 s): report pain after 1, 10, and 60 s [250 Hz]	N/A or not mentioned in study	Ice water (0–4 °C) Room temperature water	NRS: 0–5 (0 = “no pain”, 5 equal to “worst pain imaginable”)
Case M. (EEG–fMRI) (2018) [80]	Chronic pain (SCD diagnosed by hematologist) HC: (no pain) (no chronic pain conditions) Male and Female Age: Not explicitly stated Group analysis [SCD: 11 participants] [HC: 13 participants]	BrainAmp MR 64 plus (BrainProducts, Germany) 64 channels	Resting state Eyes open 20 min: outside scanner 20 min: inside scanner [1000 Hz]	MRI 3 T Siemens Magnetom Trio scanner (Erlangen, Germany) (16 channel head coil) 1 subject: 3 T Siemens Magnetom Prisma scanner (Erlangen, Germany) (20 channel head coil)	N/A or not mentioned in study	Verbal pain rating scale: 0 to 10 (0 = “no pain”, 10 = “worst pain imaginable”)
Case M. (Resting–state EEG) (2018) [80]	Chronic pain (SCD diagnosed by hematologist) HC: (no pain) (no chronic pain conditions) Male and Female Age: Not explicitly stated Group analysis [SCD: 20 participants] [HC: 14 participants]	BrainAmp MR 64 plus (BrainProducts, Germany) 64 channels	Resting state (at least 2 separate recordings) 10 min Eyes open	MRI	N/A or not mentioned in study	Verbal pain rating scale: 0 to 10 (0 = “no pain”, 10 = “worst pain imaginable”)
Nuñez-Ibero M et al. (2021) [67]	Healthy Male and Female Age: 20–40 [35 participants]	BioPac (BioPac Systems, Inc., Student Lab MP36) (Goleta, CA, USA) 6 channels	Eyes closed 2 min [500 Hz]	PPG	Computer–driven Peltier cell: applies heat at 90% of threshold (2 min)	N/A or not mentioned in study
Ocay DD et al. (2022) [62]	Chronic Pain Group (musculoskeletal pain at least once a week ≥ 3 mo), HC: (no chronic pain in past 3 months) Male and Female Age: 10–18 [Chronic Pain: 142 participants] [HC: 45 participants]	EEG headset (DSI–24, Wearable Sensing) 21 channels 10–20 system	Recording A: eyes open 2 groups: with and without moving the computerized VAS Recording B: during tonic heat Recording C: During CPT [300 Hz]	Thermal stimulation CPT	Tonic heat stimulation (warm thermode with Q–sense apparatus): 120 s CPT: 12 °C, 2 min	Douleur Neuropathique 4 (DN4) 11–point NRS: 0 = “no pain”, 10 = “worst pain imaginable” Pain Catastrophizing Scale for Children (PCS–C) Computerized VAS
G*Peier F. (2022) [68]	Trained athletes (healthy) (endurance sport ≥ 7 h a week for the last 6 months) Non–trained athletes (healthy) (sports activity ≤ 2.5 h week for the last 6 months) Male Age: 18–60 [Trained Athletes: 26 participants] [Non–Trained Athletes: 24 participants]	High Density EEG Active Two Recording Device (BIOSEMI, Amsterdam, The Netherlands) 64 channels	Baseline resting state (3 min) (randomized conditions: seated eyes open, seated eyes closed, and standing eyes closed) 2 min warm water CPT: max 4 min Recovery (with warm water & cartoons): 15 min Resting state (3 min) [1024 Hz]	QST	CPT 4 °C (duration of bearable pain: max 4 min)	11–Point NRS: 0 = “no pain”, 10 = “worst pain imaginable” CSI (assessment of CS features)
Rustamov N et al. (2021) [70]	Healthy Male and Female Age: 18–70 [12 participants]	EEG Headset (DSI 24, Wearable Sensing, San Diego, CA, USA) 24 channels 10–20 system	2 sessions (14 days apart) Baseline (10 min) CPT (2 min) Recovery (10 min) [300 Hz]	N/A or not mentioned in study	CPT (thermostat–controlled circulating cold water bath) Temperature ≥ 4°C 2 min * Device listed in paper	Verbal NRS: 0 = “no pain”, 10 = “worst pain imaginable”
Simis M, Imamura M et al. (2022a) [60]	Chronic pain (clinical diagnosis with knee osteoarthritis) Male and Female Age: 18+ [66 participants]	EGI system (Electrical Geodesics, Inc., EGI, Eugene, OR, USA) 128 channels	Resting state Eyes closed 5 min [250 Hz]	EMG CPM TMS	TMS (Magstim Rapid stimulator (The Magstim Company Limited, United Kingdom) Test stimulus: PPT (three algometry stimuli): 15-s intervals Conditioning stimulus: (contralateral hand in cold water): 10–12° C, 30 s After 30 s, do the test stimulus simultaneously [Procedure repeated with hands switched]	VAS Western Ontario and McMaster Universities Osteoarthritis Index (WOMAC)
Simis M et al. (2022b) [81]	Chronic pain (clinical & radiological diagnosis of SCI of traumatic etiology) (1 to 36 months after lesion) (“C” or “D” on American Spinal Injury Association Impairment Scale) Male and female Age: 18–65 [39 participants]	Acti–Champs (PyCorder, Brainvision LLCV) 32 channels 10–20 system	Resting state 20 min Eyes closed	CPM	Test stimulus: PPT (three algometry stimuli): 15-s intervals Conditioning stimulus: (contralateral hand in cold water): 10–12° C, 30 s After 30 s, do the test stimulus simultaneously [Procedure repeated with hands switched]	VAS
Simis M, Pacheco-Barrios K et al. (2023) [72]	Chronic pain (clinical diagnosis with knee osteoarthritis) (eligibility for IMREA rehabilitation program) Male and Female Age: 18+ [85 participants]	EGI system (Electrical Geodesics, Inc., EGI, Eugene, OR, USA) 128 channels	Resting state Eyes closed 5 min recording	CPM TMS	TMS (Magstim Rapid stimulator (The Magstim Company Limited, United Kingdom) Test stimulus: PPT (three algometry stimuli): 15-s intervals Conditioning stimulus: (contralateral hand in cold water): 10–12° C, 30 s After 30 s, do the test stimulus simultaneously [Procedure repeated with hands switched]	VAS 36 item short form (SF–36) Western Ontario and McMaster Universities Osteoarthritis Index (WOMAC)
Uygur-Kucukseymen E et al. (2020) [82]	Chronic pain (diagnosis of fibromyalgia) (American College of Rheumatology 2010 criteria) Male and female Age: 18–65 [TMS: 25 participants] [EEG: 21 participants]	EGI system (Electrical Geodesics, Inc.) (EGI, Eugene, OR, USA) 64 channels	Resting state: 10 min (5 min eyes open, 5 min eyes closed) Event–related spectral perturbations: 8 min (motor, observation, imagery tasks)	CPM TMS Surface EMG	Peltier thermode (Medoc Advanced Medical Systems, Ramat Yishai, Israel): 30 s, pain–60 temperature, right proximal volar forearm Conditioned stimulus (left hand in cold water): 10–12°C, 30 s & test stimuli simultaneously TMS (Magstim Rapid2 device): single pulse (resting motor threshold, motor evoked potentials), paired pulse (short interval cortical inhibition, intracortical facilitation)	numerical pain scale VAS (0 to 10)
van den Berg B et al. (2021) [73]	Chronic pain (failed back surgery syndrome) HC (non–failed back surgery syndrome patients) Male and Female Age: 18+ [Chronic pain: 16 participants] [HC: 17 participants)	EEG Cap (Ag/AgCl electrode cap) 64–Channel 10–20 System	Focus gaze at fixed point on wall (35–40 min) Recording during stimulus [1000 Hz]	N/A or not mentioned in study	Intra–epidermal electrical stimuli (custom made electrode w/5 micro needles with detection threshold): total of 450 stimuli applied	N/A or not mentioned in study
Vanneste S, De Ridder D. (2023) [59]	Chronic pain (Failed back surgery patients) Male and Female Age: 40–67 [15 participants]	Scalp EEG, (Neuroscan, Charlotte, NC, USA) 19 Channels 10–20 System	Eyes closed 5 min [1000 Hz]	Electromagnetic Tomography (eLORETA, available at https://www.uzh.ch/keyinst/) (accessed on 20 May 2024)	Surgical Implantation of Internal pulse generator Lamitrode 88 (Location: St. Jude Medical neurodivision, Plano, TX, USA)	VAS Pain Catastrophizing Scale
Wang H et al. (2023) [65]	Healthy Right–handed Male and female Age: 20–35 [48 participants]	Scalp Electrode (Brain Products GmbH, Munich, Germany) 64 channels 10–20 System	Eye open, relaxed, monitoring eye blinking [1000 Hz]	Skin Temperature at Thermode–Skin Interface	Thermode (CHEPS, Medco Ltd., Raman Yishai, Israel): Contact area of 27 mm diameter. Increase rate of 70 °C per sec using thermofoil, decreased 40 °C/s. using Peltier with 150 Hz sampling rate	11–point NRS: 0 = “no pain”, 10 = “worst imaginable pain”

**Table 2:** Summary of study features, methods, findings, and limitations.

Author (Year)	Study Features	Statistical Methods	Analytical Methods	Key Findings	Limitations
Barbosa SP et al. (2024) [77]	Relative power (central, frontal, and parietal regions) Demographic & clinical characteristics	Neural activity/relationships: linear regression (continuous variables), logistic regression (categorial variables) Normality assessment: univariate analysis, multivariate analysis	Spectral power: Fast Fourier transform, relative power	Decrease in pain intensity results in decreased alpha and increased theta and beta (central region) Pain effects on mood were associated with increased alpha activity (frontal and central regions)	Sample size Imbalance of biological sex in participant population
Beck B et al. (2019) [66]	Nociceptive laser–evoked potentials Non–nociceptive electrical somatosensory–evoked potentials GBO components	Two–tailed one–sample *t*-test, two–tailed paired–sample *t*- test, paired–sample *t*-test Pearson correlation tests Spearman’s rank order method	Time–domain analysis: single–trial peak N2 and P2 amplitudes Frequency–time domain: cluster–based permutation test, average power|signal detection theory: perpetual sensitivity, bias	Laser–evoked N1, N2, and P2 waves responded according to stimulus intensity (compared to electrical stimulation) Laser–evoked N2 wave showed variability in pain perception	Can not conclude that the effects are exclusive to nociceptive processing Other stimulus types not tested Time interval of the electrical stimuli may have prevented assessment of components in the A–beta lemniscal pathway.
Camargo L et al. (2024) [79]	Demographic and clinical variables CPM response, Spectral power Event–related spectral perturbation	Clinical and demographic variables: mean, percentages, standard deviations Independent and dependent variable associations: regression analysis Linear univariate (*p*–value < 0.2) & multivariate analyses	Motor task spectral power: fast Fourier transformation Event related spectral perturbation: three–cycle wavelet Hanning–tapered window, event–related desynchronization (0 ms–3000 ms) event–related synchronization (3000 ms–7000 ms) EEG spectral power analysis: relative power (frontal, central, parietal regions)	Negative correlation between pain intensity and beta band activity (frontal and parietal) Increased delta, theta, and beta activity is seen with increases in fibromyalgia duration	No control group No preliminary bi–hemispheric assessment (right hand motor execution) Cross–sectional nature
Chouchou F, Perchet C, Garcia-Larrea L. (2021) [69]	Pain perception Gamma power Alpha power EEG topography	Repeated measures ANOVA Greenhouse—Geisser correction of degrees of freedom z–value	spectral analysis: fast Fourier transform, alpha and gamma band power, region of interest (frontal, mide–central, centro–temporal, parieto–occipital) Topographical comparisons: estimator of distributional similitude	Gamma power distributions were similar between pain and grimace conditions Alpha power was more resistant to grimace muscle activity as it did not share similar oscillatory features during pain conditions	Small sample size.
Chowdhury NS et al. (2023) [75]	PAF Depression CME	Test-retest reliability: Intraclass correlation coefficient	PAF calculations: peak picking method, center of gravity	PAF reliability (excellent/moderate–good range) CME (good range) Both can serve as reliable biomarkers for sustained pain	The features were not assessed past day 5 No sham NGF injection.
De Vries M et al. (2013) [63]	Power Spectra Grand average power spectra Power distribution topography PAF	Kolmogorov–Smirnoff Test T–test for independent samples Non–parametric Mann–Whitney U test General linear model repeated measures ANOVA Mauchly’s test Greenhouse–Geisser estimation Two–sided unpaired *t*-tests nonparametric Spearman test	Power amplitudes: fast Fourier transform, averaging Grand average power spectra: averaging Peak power amplitudes: maximum value PAF: center of gravity method	PAF shifted to lower frequencies for the chronic pain group (compared to HC) Decreases in PAF correlated with longer pain duration Significant PAF differences were seen in the parietal and occipital regions between chronic pain and healthy control groups	Did not collect pain scores Differences in patient pain and profile that include comorbidity and medication.
Ding K et al. (2024) [78]	Power spectra PAF Power/power ratio, Peak frequency difference Network connectivity strength	Population mean balance: Two–sample Student’s T–test with unequal variance, Two–sample Student’s T–test Statistical significance: Bonferroni correction for multiple comparisons Statistical significance: Students *t*-test (*p* < 0.0002)	EEG Analysis: Fast Fourier transform EEG power comparison: average power spectra Absolute frequency components: mean of spectral power in frequency band PAF: geometric centroid frequency of the power spectrum Graph theory analysis	Chronic pain group (compared to controls) is associated with increased alpha power, less theta and beta power, decreased peak theta–beta frequency difference, and lower PAF During stimulation (thermal), chronic pain group alpha–theta/alpha–beta power ratios and peak theta–beta frequency difference decreased, the inverse was seen for the controls Chronic pain group showed less significant differences in network connectivity during pain stimulations compared to controls.	Small sample size Chronic pain group: possibility of differences in degree of central sensitization
Furman AJ, Thapa T et al. (2019) [76]	Sensorimotor PAF	Spearman’s rank order correlation Cronbach’s α Bonferroni corrections for multiple tests Linear support vector machines One sample T–test Bayes factor analysis Linear mixed effects model	Sensorimotor PAF: center of gravity method, averaging	Sensorimotor PAF speed at rest (no pain) was seen to be inversely correlated with nerve growth factor pain sensitivity Slower pain–free PAF was seen negatively correlated to higher pain sensitivity	Needed to know more factors like sleep quantity to have a better understanding of PAF Confined by lab Study 2 did not have an impact on the relationship between PAF and NGF pain Small study size.
Furman AJ, Prokhorenko M et al. (2020) [64]	Sensorimotor PAF	Pairwise correlations: Spearman’s rank order correlations Cronbach’s α Bonferroni corrections for multiple tests Internal validation: linear support vector machines F1 scores Paired *t*-test Bayes factor analysis Linear mixed effects model	Sensorimotor PAF: center of gravity method, averaging	Sensorimotor PAF speed at rest (no pain) was seen to be inversely correlated with pain sensitivity for both short and long durations.	The group of subjects with insensitivity to CHP were not included in the main analysis Study does not address sources and identity of PAF Limited frequency range for PAF computations.
Heitmann H et al. (2022) [61]	Dominant peak frequency Neuronal oscillation power Functional connectivity	Changes in clinical measures: frequentist and Bayesian statistics, correlation analyses, partial correlations, cluster-based permutations Sample size: sensitivity analysis with G*Power 3.1 Bonferroni method	Dominant peak frequency: local maximum, center of gravity, local center of gravity (sensorimotor), fast Fourier transformation Frequency power: source space (linearly constrained minimum variance beamforming) Functional connectivity: source space (debiased weighted phase lag index)	Pain–related disability had a positive relationship with local beta band degree (brainstem and cerebellum) Changes in theta band gEff negatively correlated with pain intensity changes and pain-related disability	Abnormal patterns in neuronal oscillations and connectivity are not exclusive to chronic pain Possible artifacts can be presented as connectivity Chronic pain in subjects mostly due to back pain, thus results cannot be generalized to other forms of chronic pain Possible effects of subjects taking medication No control group.
Kenefati G et al. (2023) [74]	Neural oscillations of Theta, Alpha, and Gamma (Medial Orbitofrontal Cortex, Anterior Cingulate Cortex, Dorsolateral Prefrontal Cortex)	Continuous variables/frequency: standard error of mean or median Behavioral data: non–parametric Mann–Whitney U test	Source–space time–frequency representations: Slepian multi–taper approach Oscillatory activity: Event related synchronization and desynchronization	CLBP group reported more pain for noxious stimuli, displayed increased mean alpha and theta power (contralateral Medial Orbitofrontal Cortex), theta (Dorsolateral Prefrontal Cortex) and high gamma (Anterior Cingulate Cortex) for 256 mN stimulus when compared to pain free controls	Small sample size Study limited to CLBP not any other form of CP Mechanical force was the same for all participants Protocol excluded temporal summation Short duration of stimuli may have limited the cortical response to only A fiber stimulation.
Levitt J et al. (2017) [71]	Power spectra Power spectral densities Coherence Functional connectivity	Mean Standard error of mean	Power spectral density: fast Fourier transform Coherence analysis	Increased pain scores seen with increased power amplitude (6–7 Hz range, frontal), decreased power/coherence (3–30 Hz, caudal), decreased coherence between Fz and C3/C4 (theta/low beta bands), increased Fz and O1 coherence (theta alpha band)	Analysis limited between 3 and 30 Hz.
Case M. (EEG–fMRI) (2018) [80]	Resting state networks Frequency analysis Connectivity EEG and fMRI power EEG–fMRI microstates,	One and two sample T–tests Demographic and clinical variables: descriptive statistics	EEG microstate analysis using power	The SCD patient group had a positive correlation between beta1 power and bilateral insula cortex activation The patient group showed reduced default mode network and executive control network activity while resting state networks had increased connectivity compared to controls	Small sample size Healthy participants as control continuing medication regularly
Case M. (Resting–state EEG) (2018) [80]	Power spectral density SVM EEG classifier EEG source imaging	Demographic and clinical variables: descriptive statistics	Power spectral density: Welch‘s method Frequency bands: average power, maximum peak, center of gravity Significance: two–sided nonparametric Wilcoxon tests, Cohen‘s method Linear model EEG power: support vector machine Source analysis: EEG cross–spectra, eLORETA,	Patient group had significantly increased theta power (prefrontal cortex, left rolandic operculum, left insula, left putamen, and the caudate nucleus) and decreased beta2 power (prefrontal cortex, anterior cingulate cortex, right superior temporal gyrus, and the right caudate nucleus) compared to the control group Greater amount of ED visits was reflected with increased theta maximum peak values.	Small patient population Patients continuing medication regularly eyes open during EEG recording ethnically diverse control group SCD could have affected neural behavior
Nuñez-Ibero M et al. (2021) [67]	Spectral entropy for EEG activity PPG Signals	Fast Fourier transform Discriminability: log10 (*p* value) Wilcoxon signed–rank test, a false–discovery–rate (FDR)/Bonferroni corrections	Spectral entropy: Shannon entropy generalization, Permutation Entropy, Multiscale Sample Entropy	Design of device for simultaneous acquisition of neural and heart responses to painful stimuli (thermal) Spectral entropy in PPG decreased (significantly) and spectral entropy for EEG increased with pain Major EEG (25–30 Hz range) and PPG (5–10 Hz) differences were seen between pain and no–pain conditions using the 1D spectral entropy	Arousal, attention, and salience were not controlled Cognitive appraisal and stimuli interval variation not assessed Limited to thermal pain & results may differ for populations with pathologies. Thermal stimulation was not randomized or repeated on the subjects
Ocay DD et al. (2022) [62]	Demographic and Clinical Characteristics Global Spectral Power Global Peak Frequencies Permutation Entropy Functional Connectivity Node Degree	Demographic and clinical measures: *t*-testing, chi–squared tests EEG features normality: Q–Q plots, Kolmogorov–Smirnov tests Pearson correlation analyses Two–way ANOVA of generalized linear mixed models Least squares means post hoc testing with Tukey corrections Least square means	Neural oscillatory activity: spectral powers (multi–taper method) Permutation entropy (probability distribution with signal motifs) Functional connectivity: weighted phase lag index, directed phase lag index	Chronic pain group exhibited age–related decrease in global theta power (resting) Increased resting global delta (resting) and beta power (resting and in pain stimulation) in the chronic pain group compared to controls Chronic pain group displayed greater global delta and theta permutation entropy during tonic heat stimulus, healthy controls did not The chronic pain group displayed a significant directed phase lag index functional connectivity decrease during the CPT in theta, alpha, and beta bands in various locations	The variables assessed could be affected by the differences in subjects‘ biophysical factors Wide range of differences in chronic pain sample Results can only infer the neurological components attributed to the observed EEG patterns Different baseline conditions.
Peier F. (2022) [68]	Features of Central Sensitization Pain Responses (resistance to pain, pain sensitivity) GABA–Dependent Inhibition	Normality: Shapiro–Wilk comparative and correlation analysis Student’s *t*-test/Mann–Whitney U test non–parametric ANOVAs Kruskal–Wallis test Correlation: Spearman’s Rho correlation coefficient and effect size	EEG spectral analysis: fast Fourier transform, frequency bands (average of frequency bins), global power spectrum (average band frequency across all electrodes)	Lower affective pain ratings were seen in athletes at pain threshold compared to non–athletes Lower beta increases with cold and pain stimulation, as well as with pain increase in non–athletes Athletes exhibit differences in pain response time, sensory pain, resistance to pain, and decreases in high beta power.	Small sample size Power was not derived appropriately Corrections were not made to accommodate for the multiple testing Age differences not controlled CPT duration maximum may have affected results Participants struggled to distinguish sensory and affective pain.
Rustamov N et al. (2021) [70]	Patterns of EEG power topographies (for pain recovery) Source generators of pain recovery Functional connectivity, phase–amplitude coupling Power spectral density Amplitude envelope correlation	Permutation tests Power spectral density source: permutation t–statistic spatiotemporal sensor map, Monte Carlo random sampling Test–retest reliability: non–parametric Spearman rank correlation	Power spectral density: Welch‘s method, fast Fourier transform Source estimation: Open–MEEG Boundary Element Method Amplitude envelope correlation: Hilbert transform, Pearson correlation coefficient Phase–amplitude coupling: Hilbert transform, Mean Vector Length approach, Canolty maps	Rebound in theta power (left fronto–central region) and increase in theta connectivity is associated with tonic pain recovery Sources of theta power over–recovery included left DLPFC, ACC and MCC, left insula and sensorimotor cortex contralaterally Tonic pain (compared to recovery): gamma amplitudes and theta/alpha phase had greater coupling.	Experimental set up did not account for differences in EEG patterns of painful and non–painful cold stimuli Small sample size Cannot translate tonic pain results to chronic pain population (needs separate testing).
Simis M, Imamura M et al. (2022a) [60]	Resting state spectral power Oscillation models (Delta, Theta, Alpha, Beta, Beta, Beta, Beta High and Low Sub–Bands)	Continuous data: mean/standard deviation or median/interquartile range Dichotomous/categorical data: frequency/percentages Shapiro–Wilk test Multivariate linear regression models Confounders: multi criteria approach	TMS bi–hemispheric average: combined resting motor threshold, cortical silent	Increase in pain intensity increased high–beta (frontocentral) power Higher relative theta (frontal, central, parietal) power was associated with lower pain ratings (WOMAC) Greater KL severity was associated with increased relatively low–beta power Delta and alpha related to high cortical inhibition	No control group Variation in patient medications and comorbidities No comparisons.
Simis M et al. (2022b) [81]	Demographic and clinical variables EEG and VAS relationship EEG and objective/subjective pain	Demographic and clinical variables: descriptive statistics Univariate analysis Logistic regression model Confounders assessment: multivariate regression Receiver operating curve for fitted logistic regressions	Power analysis: absolute power, relative power, alpha–theta and alpha–beta ratios (central, parietal, frontal)	Pain presence and higher pain levels represented with less alpha and beta power, as well as decreased peak–alpha–theta oscillations Low CPM efficiency correlates with more relative theta power	Patients continued neuroactive medication Only AIS levels C and D assessed Small sample size
Simis M, Pacheco-Barrios K et al. (2023) [72]	Neural Oscillations Neural Power	Descriptive variables: mean/standard deviation or median/interquartile range CPM model: Directed Acyclic Graph, linear and logistic univariate analyses, forward approach, purposeful selection approach	Resting EEG: absolute and relative frequency band power	Negative association between pain scores and CPM, as well as CPM and balance Relative delta power (frontal, central) and CPM have a negative relationship	No control group No corrections for multiple analyses.
Uygur-Kucukseymen E et al. (2020) [82]	Demographic and clinical variables EEG relative power Resting EEG Event-related desynchronization	Demographic and clinical variables: descriptive statistics Shapiro–Wilk univariate analysis Independent linear regression models Confounders assessment: multivariate regression models	Event-related desynchronization: calculated from central area, short time Fourier transform, Morlet wavelets, ERD calculation Power analysis: fast Fourier transform, absolute power and relative power (central, parietal, frontal) Resting motor threshold Motor evoked potentials: mean peak-to-peak amplitudes Short interval cortical inhibition/intracortical facilitation: motor evoked potential ratio	Higher pain levels resulted in lower alpha and beta power (central region) as well as lower theta and delta ERD (central region) Smaller theta and delta ERD positively correlated with short interval cortical inhibition and CPM efficiency, and inversely related to intracortical facilitation	ERD calculation based on two types of spectrum analysis (non–homogenous calculation) Small sample size No control group
van den Berg B et al. (2021) [73]	Psychophysical features Random forest classification	Psychophysical features: generalized linear model	Neural features: N1 and P1 latencies, linear model Random forest classification	Logarithm of psychophysical slopes and detection thresholds, trial number and second–pulse amplitude effects, detection rate, standard deviation of reaction time were significantly different between the FBSS and control groups Depending on the features, the random forest classifier had an accuracy between 0.65 and 0.78	Larger variation made it difficult to detect a nociceptive stimulus, little significant difference between averaging or computing standard deviation of the EEG, and only using EEG resulted in lower accuracy.
Vanneste S, De Ridder D. (2023) [59]	Region of interest, Lagged phase coherence, Granger causality, Cross–Frequency Coupling.	Repeated measures ANOVA (*p* < 0.05)	sLORETA and eLORETA	Decrease in pain intensity and suffering from SC stimulation shows a decrease in alpha and beta oscillations (dACC), theta (pgACC)	Low sample size Correction methods decrease statistical power.
Wang H et al. (2023) [65]	Contact Heat Evoked Potentials (CHEP) Low–Frequency Components (LFC) Neural Oscillations (Alpha and Beta Frequencies) Neural Origins Phase–Amplitude Coupling Mediation Effects	2–way repeated measures ANOVA T–tests	Low frequency component: averaged LFC signals, spline interpolation Time–frequency analysis: Windowed Fourier transform, Averaging Source: LORETA, Classical LORETA Analysis Recursively Applied, Multiple Source Beamformer Phase–amplitude coupling: envelope–to–signal correlation method Multi–level mediation analysis: 2–path and 3–path multilevel mediation analyses	N2 and P2 amplitudes were greater in response to more intense stimulation Alpha–ERD duration and amplitude were associated with stimulus duration LFC sources: insula (contralateral and ipsilateral to stimulation site), ACC Alpha–ERD Sources: primary sensorimotor cortex (contralateral and ipsilateral to stimulation site) Alpha–ERD amplitude coupled with LFC phase	Lack of perdition with time intervals Small differences in time intervals Low signal to noise ratio for higher frequencies and spatial resolution No control condition in different stimulus types.

**Table 3:** Major findings for chronic and experimentally induced pain.

Feature	Chronic Pain	Experimentally Induced Pain
Peak Alpha Frequency (PAF)	Evident PAF abnormalities Slow or decreased PAF	Evident PAF abnormalities Slow or decreased PAF
Prefrontal Control	Reduced ability to regulate pain intensity or emotional responses	Intact functional connectivity; returns to baseline after stimulation
Emotional Networks	Increased coupling No studies on emotional aspects	Minimal to no coupling No studies on emotional aspects
Default Mode Network (DMN)	Increased connectivity, particularly in medial prefrontal cortex (mPFC) and posterior cingulate cortex (PCC) Disrupted balance	Minimal to no long-term changes in DMN connectivity Transient suppression during stimuli

## Abbreviations

International Association for the Study of Pain (IASP), Visual Analog Scale (VAS), Spinal Cord Stimulation (SCS), electroencephalogram (EEG), functional Magnetic Resonance Imaging (fMRI), event-related potentials (ERPs), Preferred Reporting Items for Systematic Reviews and Meta-Analysis (PRISMA), “Population, Intervention, Comparison, Outcome” (PICO), internal pulse generator (+IPG), healthy control (HC), pregenual anterior cingulate cortex (pgACC), dorsal anterior cingulate cortex (dACC), somatosensory cortex (SSC), conditioned pain modulation (CPM), knee osteoarthritis (KOA), Western Ontario and McMaster Universities Osteoarthritis Index (WOMAC), Berg Balance Scale (BBS), interdisciplinary multimodal pain therapy (IMPT), global efficiency (gEff), chronic pancreatitis (CP), peak alpha frequency (PAF), quantitative sensory testing (QST), German Research Network on Neuropathic Pain (DFNS), pain pressure threshold (PPT), phasic heat pain (PHP), Contact Heat Evoked Potential Stimulator (CHEPS), numerical rating scale (NRS), capsaicin heat pain (CHP), gamma-band oscillations (GBO), photoplethysmography (PPG), gamma-aminobutyric acid (GABA), central sensitization (CS), central sensitization inventory (CSI), failed back surgery syndrome (FBSS), fibromyalgia (FM), sickle cell disease (SCD), chronic lower back pain (CLBP), dorsolateral prefrontal cortex (dlPFC), anterior cingulate cortex (ACC), nerve growth factor (NGF), transcranial stimulation (TMS), corticomotor excitability (CME), electromyography (EMG), laser-evoked potentials (LEPs), cold pressor test (CPT), magnetoencephalography (MEG), positron emission tomography (PET), functional connectivity (FC), phase locking value (PLV), mutual information (MI), cross-frequency coupling (CFC), default mode network (DMN), medial prefrontal cortex (mPFC), posterior cingulate cortex (PCC).

## Appendix A

**Table A1:** Study risk of bias assessment (CPT: cold pressor test; HPT: heat pain threshold; NGF: nerve growth factor).

Source	Criteria for Subject Selection	Use of Any Medication	Quality Criteria from Newcastle–Ottawa Scale			Total Stars (Up to 10 Stars)
			Selection (Up to 4 Stars)	Comparability Between Groups (Up to 2 Stars)	Exposure (Up to 4 Stars)	
Barbosa SP et al. (2024) [77]	PDQ Pre-existing dataset from Zolezzi et al., (2023) [83]	Partially Yes (*n* = 18 centrally acting drugs, cannabidiol derivatives, nonsteroidal anti-inflammatory (NSAID) drugs) No (*n* = 12)	4	2	3	9
Ding K et al. (2024)** [78]**	Study-specific inclusion/exclusion criteria (chronic pain versus healthy control)	No	4	2	3	9
Camargo et al. (2024)** [79]**	Study-specific inclusion/exclusion criteria (chronic pain and stimulated pain)	No substance abuse within past six months and use of opioids in large doses (more than 30 mgofoxycodone/hydrocodone or 7.5 mg of hydromorphone or equivalent)	2	1	3	6
Chowdhury NS et al. (2023)** [75]**	Inclusion criteria: PREDICT study participants Study-specific exclusion criteria	No	4	2	3	9
Vanneste S, De Ridder D. (2023)** [59]**	Study-specific inclusion/exclusion criteria	Yes. Opioids and antiepileptic drugs	2	2	2	6
Kenefati G et al. (2023)** [74]**	Study-specific inclusion/exclusion criteria	No benzodiazepine	3	2	3	8
Wang H et al. (2023)** [65]**	Study-specific inclusion/exclusion criteria (healthy control and noxious contact heat stimuli)	Not informed	4	2	3	9
Simis M, Pacheco-Barrios K et al. (2023)** [72]**	Study-specific inclusion/exclusion criteria (only chronic knee OA pain patients); No healthy control group	Yes, but not specified	2	2	2	6
Simis M et al. (2022a)** [60]**	Study-specific inclusion/exclusion criteria (only chronic knee OA pain patients) No healthy control group	Yes, but not specified	2	2	2	6
Simis M et al. (2022b)** [81]**	Study-specific inclusion/exclusion criteria (chronic pain only)	Yes, neuroactive medication	2	2	2	6
Heitmann H et al. (2022)** [61]**	Study-specific inclusion/exclusion criteria (only chronic pain patients) No healthy control group	Yes, except for regular benzodiazepine medication	2	2	2	6
Ocay DD et al. (2022)** [62]**	Study-specific inclusion/exclusion criteria (chronic pain patients versus healthy control group)	Not informed	3	2	3	8
Peier F. (2022)** [68]**	Study-specific inclusion/exclusion criteria (healthy highly trained athletes versus healthy non-trained non-athletes) CPT	No	4	2	3	9
Nuñez-Ibero M et al. (2021)** [67]**	Study-specific inclusion/exclusion criteria (heat pain threshold (HPT)) Control group.	No	4	2	3	9
Chouchou F, Perchet C, Garcia-Larrea L. (2021)** [69]**	Study-specific inclusion/exclusion criteria. (stimulated pain by CPT versus stereotyped grimaces mimicking those evoked by pain)	No, except for contraceptive pills	4	2	3	9
Rustamov N et al. (2021)** [70]**	Study-specific inclusion/exclusion criteria (stimulated pain by CPT versus baseline)	No analgesics, including non-steroidal anti-inflammatory drugs (NSAIDs) or acetaminophen, for at least five drug half-lives prior	4	2	3	9
van den Berg B et al. (2021)** [73]**	Study-specific inclusion/exclusion criteria (failed back surgery patients versus healthy control group)	Yes, not specified	4	2	3	9
Furman AJ, Prokhorenko M et al. (2020)** [64]**	Study-specific inclusion/exclusion criteria (stimulated pain by phasic heat pain and capsaicin heat pain models)	No	4	2	3	9
Uygur-Kucukseymen E et al. (2020)** [82]**	Study-specific inclusion/exclusion criteria (chronic pain)	No opiate use in large doses	2	2	2	6
Furman AJ, Thapa T et al. (2019)** [76]**	Study-specific inclusion/exclusion criteria (stimulated pain by repeated intramuscular injection of nerve growth factor (NGF))	No	4	2	2	8
Beck B et al. (2019)** [66]**	Study-specific inclusion/exclusion criteria (stimulated pain)	No	4	2	3	9
Case M (2018)** [80]**	Study-specific inclusion/exclusion criteria (chronic pain versus healthy control)	Yes, not specified	4	2	4	10
Levitt J et al. (2017)** [71]**	Study-specific inclusion/exclusion criteria (stimulated pain)	No	4	2	3	9
De Vries M et al. (2013)** [63]**	Study-specific inclusion/exclusion criteria Chronic pain versus healthy controls	Yes, analgesics, including opioids, and centrally acting medication No alcohol	4	2	3	9
